# 4,5-Diazafluorene and 9,9’-Dimethyl-4,5-Diazafluorene as Ligands Supporting Redox-Active Mn and Ru Complexes

**DOI:** 10.3390/molecules25143189

**Published:** 2020-07-13

**Authors:** Wade C. Henke, Julie A. Hopkins, Micah L. Anderson, Jonah P. Stiel, Victor W. Day, James D. Blakemore

**Affiliations:** Department of Chemistry, University of Kansas, 1567 Irving Hill Road, Lawrence, KS 66045, USA; whenke@ku.edu (W.C.H.); jhop0910@ku.edu (J.A.H.); micah.l.anderson@students.jsums.edu (M.L.A.); jonah.stiel@ku.edu (J.P.S.); vwday@ku.edu (V.W.D.)

**Keywords:** manganese tricarbonyl, ruthenium, electrochemistry, 4,5-diazafluorene, 9,9’-dimethyl-4,5-diazafluorene

## Abstract

4,5-diazafluorene (daf) and 9,9’-dimethyl-4,5-diazafluorene (Me_2_daf) are structurally similar to the important ligand 2,2’-bipyridine (bpy), but significantly less is known about the redox and spectroscopic properties of metal complexes containing Me_2_daf as a ligand than those containing bpy. New complexes Mn(CO)_3_Br(daf) (**2**), Mn(CO)_3_Br(Me_2_daf) (**3**), and [Ru(Me_2_daf)_3_](PF_6_)_2_ (**5**) have been prepared and fully characterized to understand the influence of the Me_2_daf framework on their chemical and electrochemical properties. Structural data for **2**, **3**, and **5** from single-crystal X-ray diffraction analysis reveal a distinctive widening of the daf and Me_2_daf chelate angles in comparison to the analogous Mn(CO)_3_(bpy)Br (**1**) and [Ru(bpy)_3_]^2+^ (**4**) complexes. Electronic absorption data for these complexes confirm the electronic similarity of daf, Me_2_daf, and bpy, as spectra are dominated in each case by metal-to-ligand charge transfer bands in the visible region. However, the electrochemical properties of **2**, **3**, and **5** reveal that the redox-active Me_2_daf framework in **3** and **5** undergoes reduction at a slightly more negative potential than that of bpy in **1** and **4**. Taken together, the results indicate that Me_2_daf could be useful for preparation of a variety of new redox-active compounds, as it retains the useful redox-active nature of bpy but lacks the acidic, benzylic C–H bonds that can induce secondary reactivity in complexes bearing daf.

## 1. Introduction

2,2’-bipyridyl (bpy) is among the most ubiquitous ligands in inorganic and organometallic chemistry. As a chelating ligand, bpy often binds to transition metals in a bidentate (κ^2^) mode and can support a variety of compounds with useful photophysical, redox, and/or catalytic properties [1,2,3,4,5,6,7]. Metal complexes and catalysts bearing bpy-type ligands can be tuned by appending electron-donating groups (EDG) and electron-withdrawing groups (EWG) to the bpy ligand; such groups primarily modulate the π-accepting ability of the conjugated framework and, to a lesser extent, the σ-donating ability of the nitrogen donor atoms. For example, we have recently used 4,4’-disubstituted-2,2’-bipyridyl (^R^bpy) ligands to tune the photophysical properties and light-induced reactivity of Mn(CO)_3_X(^R^bpy) complexes [8] as well as to modulate the accessible pathways and efficiency of dihydrogen production by [Cp*Rh] complexes bearing ^R^bpy ligands [9]. Such modifications have also been used to tune catalysis of carbon dioxide (CO_2_) reduction to carbon monoxide (CO) by [Re(CO)_3_] and [Mn(CO)_3_] complexes [10,11]. With these observations and many others from the field, ^R^bpy ligands have been found to be uniquely suited to systematic investigation of transition metal complexes. Furthermore, the wide range of accessible ^R^bpy ligands makes them attractive for efforts in rational design of new metal complexes and molecular catalysts. 

Ligands based upon 4,5-diazafluorene (daf) have several features in common with the workhorse ^R^bpy ligands, and thus offer a notable alternative for development of new metal complexes and catalysts [1]. In particular, both daf and bpy have 12e^−^ π systems and both commonly bind to metals in a κ^2^ fashion. However, daf is distinguished from bpy by its more rigid structure, attributable to the linking inter-ring sp^3^-hybridized carbon present in the fused five-membered ring. Photochemical studies of metal complexes supported by daf and bpy have mapped the importance of these features, including involvement of the daf π-system in metal-to-ligand charge transfer behavior [12,13]. Furthermore, the constrained chelate angle of daf has been implicated in giving rise to more significant excited-state reactivity than that encountered for bpy [14]. 

Unfunctionalized daf features two doubly benzylic C–H bonds at the 9-position, opening further possibilities for ligand-centered acid/base reactivity that cannot occur with simple 2,2’-bipyridyl derivatives. Along these lines, Song and co-workers have explored the coordination chemistry of daf and substituted diazafluorenes, including significant work aimed at leveraging this unique acid/base chemistry [15]. In their work, Song and co-workers have found that the acidic C–H bonds of daf can undergo deprotonation that results in follow-up reactivity [16,17,18,19,20]. More broadly, Stahl [21,22,23] and several other groups [24,25,26] have developed a number of catalyst systems supported by diazafluorene ligands. In all these cases, daf and its derivatives seem to play a decisive role in enabling unique chemistry, confirming the usefulness of the ligands as a counterpoint to the more common ^R^bpy family. 

As we have found in our own work that redox-active compounds and catalysts can be readily tuned by substituent effects with ^R^bpy ligands [8,9], 4,5-diazafluorene-based ligands could be useful in modulating the structural, electronic, and electrochemical properties of redox-active compounds more commonly supported by ^R^bpy derivatives. In particular, the coordination chemistry of the ligand 9,9’-dimethyl-4,5-diazafluorene (Me_2_daf) has received less attention than it deserves [23], as this ligand avoids the acidic C–H bonds present in daf that can readily engage in non-innocent behavior. Furthermore, reliable methods from Schmidt and co-workers [27] and Tetsuya and co-workers [28] are available for preparation of Me_2_daf, encouraging further exploration of its chemistry. 

**Chart 1:**
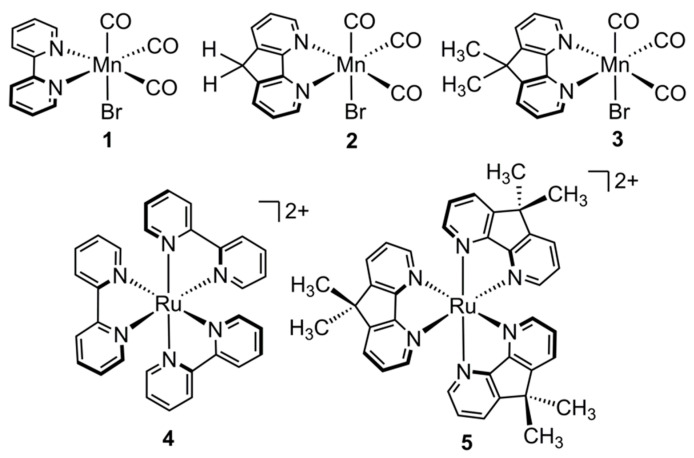
Manganese tricarbonyl and ruthenium complexes supported by bpy, daf, and Me_2_daf discussed in this study.

Here, we now report the synthesis, characterization, and electrochemical properties of Mn(CO)_3_Br(daf) (**2**), Mn(CO)_3_Br(Me_2_daf) (**3**), and [Ru(Me_2_daf)_3_](PF_6_)_2_ (**5**), and compare their properties to the more common analogues Mn(CO)_3_(bpy)Br (**1**) and [Ru(bpy)_3_]^2+^ (**4**), respectively (see Chart 1 for structures of all compounds). We find that the use of daf and Me_2_daf ligands in the complexes leads to unique spectroscopic features in the NMR and electronic absorption spectra, as well as a characteristic shift in the C–O vibrational frequencies found in the infrared (IR) spectra of **2** and **3** compared to that of **1**. Consistent with these spectroscopic observations, results from single-crystal X-ray diffraction analysis of **2**, **3**, and **5** reveal wider chelate angles and elongated M-N bond lengths in comparison with the analogous bpy complexes. The new complexes exhibit electrochemical profiles that are akin to those of their bpy analogues, confirming the similar redox-active natures of bpy, daf, and Me_2_daf. However, related tests show that complexes **2** and **3** are not catalysts for the reduction of CO_2_ to CO, contrasting with the robust catalytic behavior of **1** [4]. Taken together, these results suggest that Me_2_daf is an attractive ligand for the development of new coordination compounds for use in studies of redox chemistry and catalysis.

## 2. Results and Discussion

### 2.1. Synthesis and NMR Characterization of Complexes ***2**, **3**, and **5***

**Scheme 1:**
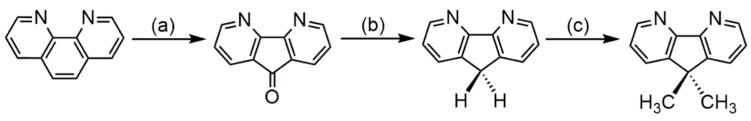
The synthetic pathway for the generation of daf and Me_2_daf. (**a**) 1. KOH, KMNO_4_; H_2_O, 16 h, 100 °C (**b**) NH_2_NH_2_·H_2_O; diethylene glycol, 170 °C, (**c**) 1. tBuOK 2. MeI; THF, −10 °C to rt.

In order to synthesize the new compounds **2**, **3**, and **5**, we first prepared the daf and Me_2_daf ligands according to literature procedures starting from 1,10-phenanthroline (phen). Oxidation of the unique olefinic functionality within phen results in the production of 4,5-diazafluoren-9-one (dafone); Wolf-Kishner reduction of dafone with hydrazine hydrate results in the generation of the daf [27]. To generate Me_2_daf, we initially attempted deprotonation of the daf methylene protons using *n*-butyllithium, but in our hands this resulted in decomposition. Instead, we utilized a milder, sterically hindered base, potassium *tert*-butoxide (tBuOK), to deprotonate daf, followed by the addition of iodomethane, to generate the anticipated Me_2_daf ligand [28] (Scheme 1). 

With the desired ligands in hand, we next moved to prepare **2** and **3** with synthetic chemistry developed earlier by Wrighton, Meyer, and others for related bpy and phen derivatives [29,30,31]. Suspension of the appropriate ligand with Mn(CO)_5_Br in diethyl ether at 38 °C results in the generation of complexes **2** and **3** in moderate yields, 62% and 73%, respectively. Previously, Cherry and co-workers have reported the synthesis of the complex [Ru(daf)_3_](PF_6_)_2_ to examine its structural properties and photophysical properties [12]. By adapting this literature procedure, the Me_2_daf analogue of [Ru(bpy)_3_]^2+^ could be prepared in a relatively low yield of ca. 17%. As an aside, we anticipate that the modest yield is likely due to differences in solubility between [Ru(daf)_3_](PF_6_)_2_ and **5** engendered by the methyl groups of Me_2_daf. Notably, all the compounds in this study were found to be acutely light sensitive and were handled in the dark or under red light to the extent possible. Following successful generation of the complexes they were each fully characterized (see Experimental Section and Figures S1–S9). 

**Figure 1:**
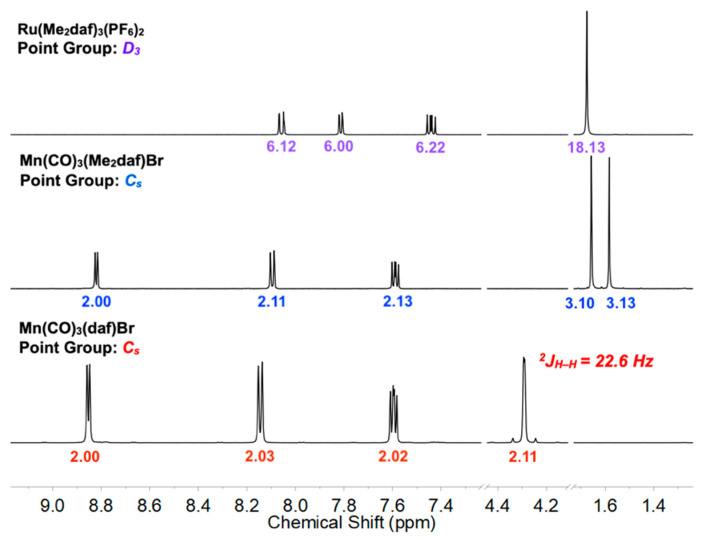
Partial ^1^H NMR spectra of **2** (bottom), **3** (middle), and **5** (top) in CD_3_CN. Peak integrations are given beneath each resonance or multiplet in colored text.

To begin characterization of the newly synthesized complexes, we turned to nuclear magnetic resonance (NMR) spectroscopy. Complexes **2**, **3**, and **5** each exhibit three resonances in the aromatic region of their ^1^H-NMR spectra with splitting patterns arising from ^3^*J*_H–H_ and ^4^*J*_H–H_ coupling; these signals correspond to the hydrogen atoms on the pyridyl rings of the daf and Me_2_daf ligands coordinated to their respective Mn and Ru centers (see Figure 1). Notably, complexes **2**, **3**, and **5** exhibit unique resonances for their daf-methylene and Me_2_daf-methyl protons. While complexes **2** and **3** exhibit C_s_ symmetry in solution, complex **5** shows *D_3_* symmetry. Correspondingly, the six methyl groups belonging to the three Me_2_daf ligands coordinated to the Ru center give rise to a singlet at 1.68 ppm (integrating to 18 H) confirming the successful preparation of complex **5**. The assignment of *D*_3_ symmetry suggests that complex **5** is chiral and thus should be present as a 50:50 racemic mixture (of Δ and Λ isomers; *vide infra*). However, enantiomers have identical chemical and physical properties and thus we observe no additional resonances in the NMR spectra for the material isolated here.

Considering the change in symmetry from *D_3_* for **5** to *C_s_* symmetry for **2** and **3**, unique NMR resonance in the latter two cases can be readily interpreted. Complex **2** possesses *C_s_* symmetry in solution and, as a result, the chemical environment of the two protons on the methylene bridge (9-position) become chemically distinct from each other and are diastereotopic. This results in a distinctive signal centered at 4.29 ppm. The geminal coupling between the two methylene protons on daf might be anticipated to give rise to two unique doublets. However, when the frequency of the coupling constant (^2^*J* = 22.6 Hz) is on the same order of magnitude as the chemical shift difference (25 Hz) between the two expected resonances, the usual one-to-one value for the resonance intensities is not observed [32,33]. Instead, a multiplet with intense inner peaks and weaker outer peaks is obtained, providing a diagnostic signal for the generation of complex **2** (in general, a phenomenon known in the field as “roofing”). The identity of this signal is further confirmed by ^13^C-distortionless enhancement polarization transfer (DEPT-135) and 2D ^1^H-^13^C heteronuclear single quantum coherence (HSQC) NMR techniques (see Figures S10–S11). Similarly, complex **3** exhibits *C_s_* symmetry in solution; the methyl groups on the apical carbon are diastereotopic, with one methyl oriented toward the axial CO ligand and the other oriented toward the bromide ligand. The difference in the chemical environment between the methyl group protons gives rise to the anticipated diastereotopic resonances; these were observed using ^1^H and ^13^C NMR, providing two signals for the protons (δ 1.58 and 1.66 ppm, each integrating to 3 H) and two signals for the carbons (δ 24.4 and 25.3 ppm), confirming the expected structure of **3** in solution. 

### 2.2. Electronic Absorption, IR, and X-ray Diffraction Studies 

**Figure 2:**
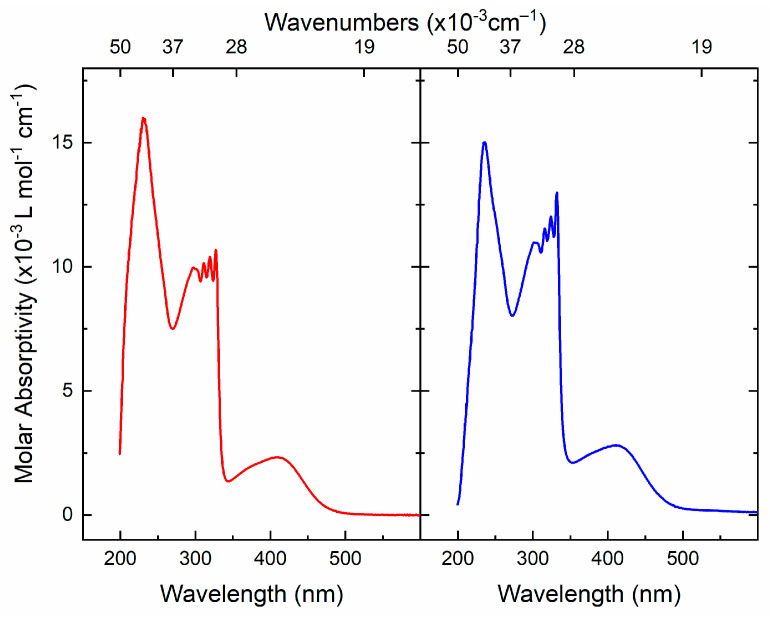
Electronic absorption spectra for **2** (left panel) and **3** (right panel) in MeCN.

Complexes **1**–**5** are all highly colored and thus we next turned to electronic absorption (EA) spectroscopy. The EA spectrum for complex **5** exhibits a strong transition at 445 nm with a molar absorptivity of 13,000 M^−1^cm^−1^ (see Figures S17–S25). The value of the molar absorptivity and the remarkable similarity of the spectrum to that of complex **4** enables assignment of this transition as a metal-to-ligand charge transfer (MLCT) [34,35]. This assignment is also consistent with known ability of daf ligands to enable visible-light induced charge transfer events at transition metal centers, similar to what is observed for complexes bearing bpy [12,13]. The observation of an MLCT transition for **5** supported by Me_2_daf is also reasonable, since the two methyl groups installed at the 9 position of daf do not perturb the conjugated system of the two aromatic rings. The EA spectra for complexes **2** and **3** reveal transitions in the visible region at 410 nm and 411 nm with molar absorptivities of 2200 M^−1^cm^−1^ and 3300 M^−1^cm^−1^, respectively (see Figure 2). Notably, these EA spectra are very similar to complex **1** [8], and based on this similarity, we are confident that these transitions can also be attributed to MLCT events. 

However, a distinguishing feature of the EA spectra of complexes **2** and **3** compared to that of complex **1** is the presence of four, relatively narrow absorptions in the UV region between 250 and 350 nm. Based on their wavelengths and molar absorptivities, these absorptions can be assigned as π-π* excitations displaying marked vibronic coupling. Such vibronic coupling has previously been observed for titanium complexes bearing diazafluorenide ligands [36], suggesting that vibronic coupling may be a common feature of the spectral profiles ligated by daf or substituted diazfluorenes. As expected, the spacing between the sharp transitions is uniform in a progression from approximately 700 cm^−1^ to 900 cm^−1^. This common observation for **2** and **3** suggests that the vibronic couplings engendered by daf and Me_2_daf are similar in these compounds. Based on this rich spectroscopic profile, we anticipate that **2** and **3** may behave differently in the presence of light than the bpy analogue **1**, encouraging further work in the future to gain insight into how these complexes behave following exposure to visible and/or UV light [8].

**Figure 3:**
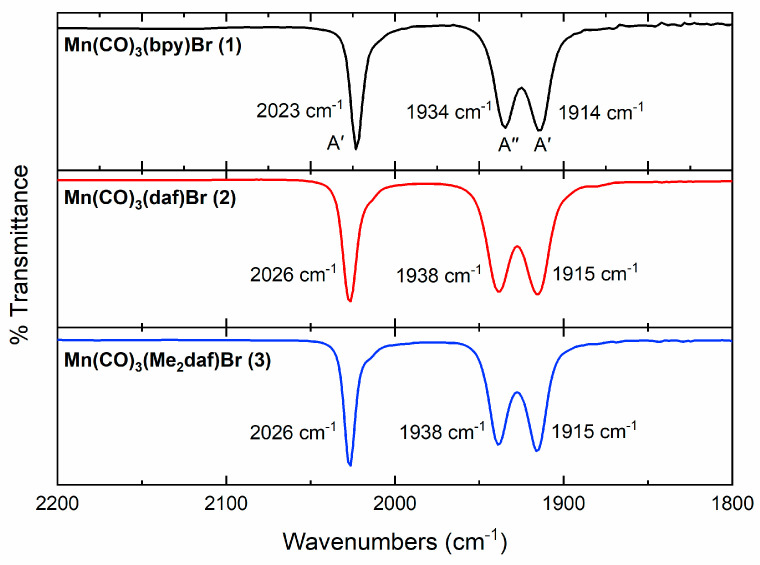
FTIR spectra of **1**–**3** in THF solution.

The IR spectra of complexes **1**, **2**, and **3** confirm that the starting material, Mn(CO)_5_Br (associated with absorption bands at 2004 cm^−1^, 2046 cm^−1^, and 2083 cm^−1^) was consumed during the synthetic reactions and is not present in the products. The *C_s_* symmetry of a *fac*-tricarbonyl complex is expected to give rise to three distinct C–O stretches in IR spectra based on group theory analysis. Upon examination of the experimental data, a three-band spectrum is observed and confirms the expected *fac*-tricarbonyl geometry for the complexes in THF solution (see Figure 3). The complexes have rather similar C–O stretching, likely a consequence of the similar environment at Mn in all three cases. In particular, C–O stretching frequencies are primarily affected by π-bonding effects, and as the π-character of bpy, daf, and Me_2_daf are not significantly different, a large shift in the vibrational frequencies for the CO ligands among **1**, **2**, and **3** is not expected. On the other hand, the modest shifts that are observable likely arise from the increased chelate bite angle of daf (**2**, 82.14(10)°) and Me_2_daf (**3**, 82.2(3)°) compared to bpy (78.80(7)°, *vide infra*) [37]. As a result of the increased bite angle, the σ-donor power of the nitrogen donor atoms to the manganese center should be decreased, resulting in a correspond increase in the C–O stretching frequency because of decreased Mn-to-CO backbonding. In accordance with this model, the vibrational frequencies for **2** and **3** are virtually identical, confirming that the addition of distal methyl groups at the ligand 9 position does not substantially perturb the structure of Me_2_daf in comparison with daf. To gain further structural insights into the properties of the new compounds, we next turned to X-ray diffraction (XRD) analysis. 

**Figure 4:**
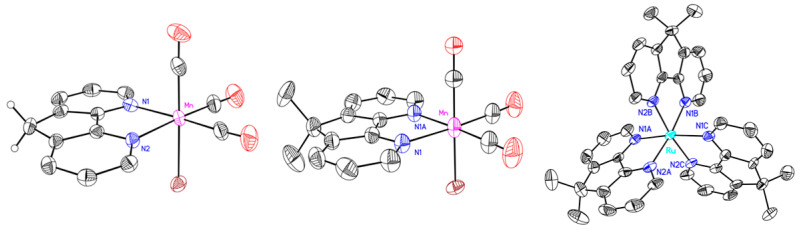
Solid-state structures of **2** (left), **3** (middle), and **5** (right, from structure q36k). Displacement ellipsoids are shown at 50% probability level. Hydrogen atoms (except H14A and H14B for **2**) and outer sphere hexafluorophosphate counteranions and disordered co-crystallized solvent (for **5**, from structure q36k) are omitted for clarity.

Vapor diffusion of diethyl ether into a concentrated THF solution of **2**, or vapor diffusion of diethyl ether into a concentrated acetonitrile (MeCN) solution of **3**, results in yellow crystals suitable for single crystal X-ray diffraction studies (see Figure 4). The results confirm the expected *fac*-geometry of the complexes with two equatorial CO ligands, an axial CO ligand, an axial bromide, and a κ^2^-daf ligand surrounding the manganese center. Although this is the first example of a formally Mn(I) complex chelated by daf or Me_2_daf, the octahedral geometries of **2** and **3** resemble those of the analogous Mn(CO)_3_(^R^bpy)Br complexes [8,37]. However, there is a significant increase in the diimine ligand bite angle for complexes **2** (82.14(10)°) and **3** (82.2(3)°) compared to **1** (78.80(7)°, *vide supra*). Additionally, the average Mn–N distances for **2** and **3** are significantly longer than those of complex **1** (2.118(4) Å and 2.109(5) Å vs. 2.047(3) Å, respectively) [37]. This is attributable to the rigid polycyclic structure of the daf framework, enforced by the inter-ring methylene group at the 9 position, which presumably drives poorer orbital overlap between the metal center and the ligand in the cases of **2** and **3**, and results in an overall increase in the M–N bond distances. 

Complex **5** is chiral and possesses *D_3_* symmetry in solution on the basis of NMR spectra (*vide supra*). No measures were taken to obtain enantiomerically pure material, and thus we isolated **5** as the 50:50 racemic mixture of delta (Δ) and lambda (Λ) isomers. Vapor diffusion of pentane into a concentrated acetone solution and vapor diffusion of pentane into a concentrated 50:50 acetone/THF solution resulted in two separate sets of orange crystals of **5** that were suitable for single-crystal XRD studies (see Figure 4). These two structures, named v74e and q36k respectively, both provide data confirming the successful synthesis of the [Ru(Me_2_daf)_3_]^2+^ core and reveal bond distances and angles that are within error of each other (see the Supporting Information, Table S3 and S4 for comparisons). On the other hand, q36k represents a higher quality structure and will be discussed here. As expected, the average chelate angle (N-Ru-N) and corresponding average Ru–N distance for complex **5** (data from q36k) are larger than in the case of the famous [Ru(bpy)_3_]^2+^ (82.9(3)° vs. 78.9(2)°; 2.117(13) Å vs. 2.063(6) Å) [38,39,40]. Gratifyingly, these values align with structural data previously available for [Ru(daf)_3_]^2+^, confirming that use of daf or Me_2_daf to form homoleptic Ru(II) complexes results in wider chelate angles and longer Ru–N distances in both cases [41].

Overall, observing the increased bite angles of the daf and Me_2_daf ligands in complexes **2**, **3**, and **5** was gratifying, since these changes should influence the electronic properties and reactivity at the metal centers in comparison with their bpy-supported analogues. Therefore, we next turned to electrochemical methods to probe the redox properties of these systems, with a particular focus on identifying features that distinguish the daf and Me_2_daf compounds from their bpy-supported analogues.

### 2.3. Electrochemical Studies

**Figure 5:**
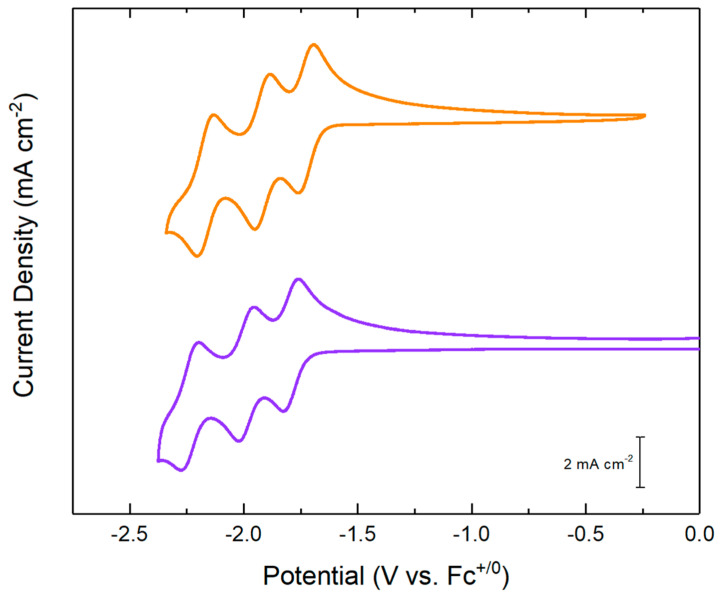
Cyclic voltammetry of **4** (orange) and **5** (purple) in MeCN solution with 0.1 M TBAPF_6_ supporting electrolyte (working electrode: highly oriented pyrolytic graphite; pseudo-reference electrode: Ag^+/0^; counter electrode: Pt wire). Ferrocene was used as an internal potential reference.

Initial cyclic voltammetry experiments were performed with **4** and **5** to interrogate how Me_2_daf behaves under electrochemical conditions in comparison to bpy (see Figure 5). As one scans cathodically, the cyclic voltammetry of the parent bpy-complex **4** exhibits three quasi-reversible reductions centered at −1.73 V, −1.92 V, and −2.17 V respectively (all potentials are quoted versus ferrocenium/ferrocene, denoted Fc^+/0^). Based on previous electrochemical studies, these reductive features can be confidently assigned to ligand-centered events; the complex is progressively reduced from [Ru^II^(bpy)_3_]^2+^, to [Ru^II^(bpy)_2_(bpy^−^)]^+^, to [Ru^II^(bpy)(bpy^−^)_2_], and finally to [Ru^II^(bpy^−^)_3_]^−^ [42,43,44]. This rich manifold of accessible ground-state reductions for **4** highlights the redox non-innocence of the bpy ligand; redox non-innocent ligands continue to grow in popularity [1,4,45,46] because of their wide-ranging applications in redox chemistry and small-molecule activation. 

We were excited to find that the cyclic voltammetric profile of **5** is remarkably similar to that of **4**. As scanning cathodically with **5** reveals three quasi-reversible reductions at −1.79 V, −1.99 V, and −2.24 V, respectively; each is centered at a slightly more negative potential than the corresponding event associated with bpy-complex **4**. The more negative reduction potentials likely arise from the inductive effect of the additional fused five-membered ring and methyl groups of Me_2_daf, resulting in a structure that is overall more electron-rich and slightly increasing the reduction potentials associated with Me_2_daf-centered reductions of **5**. Based on the electronic similarities of bpy and Me_2_daf, we can reliably implicate redox non-innocence of the Me_2_daf ligand as giving rise to the manifold of reductions observed for **5**, similar to the case of bpy in **4**. Considering this situation, we anticipate that **5** may have significant photochemical reactivity, and might serve as a useful photosensitizer in future work. 

Consistent with the ligand-centered nature of the reductive events measured for **4** and **5**, the difference in the bite angle between Me_2_daf and bpy does not strongly affect the reductive cyclic voltammetry of these compounds. However, confirmation that Me_2_daf behave as a redox-active ligand suggests that similar processes may be accessible in the tricarbonyl compounds **2** and **3**.

**Figure 6:**
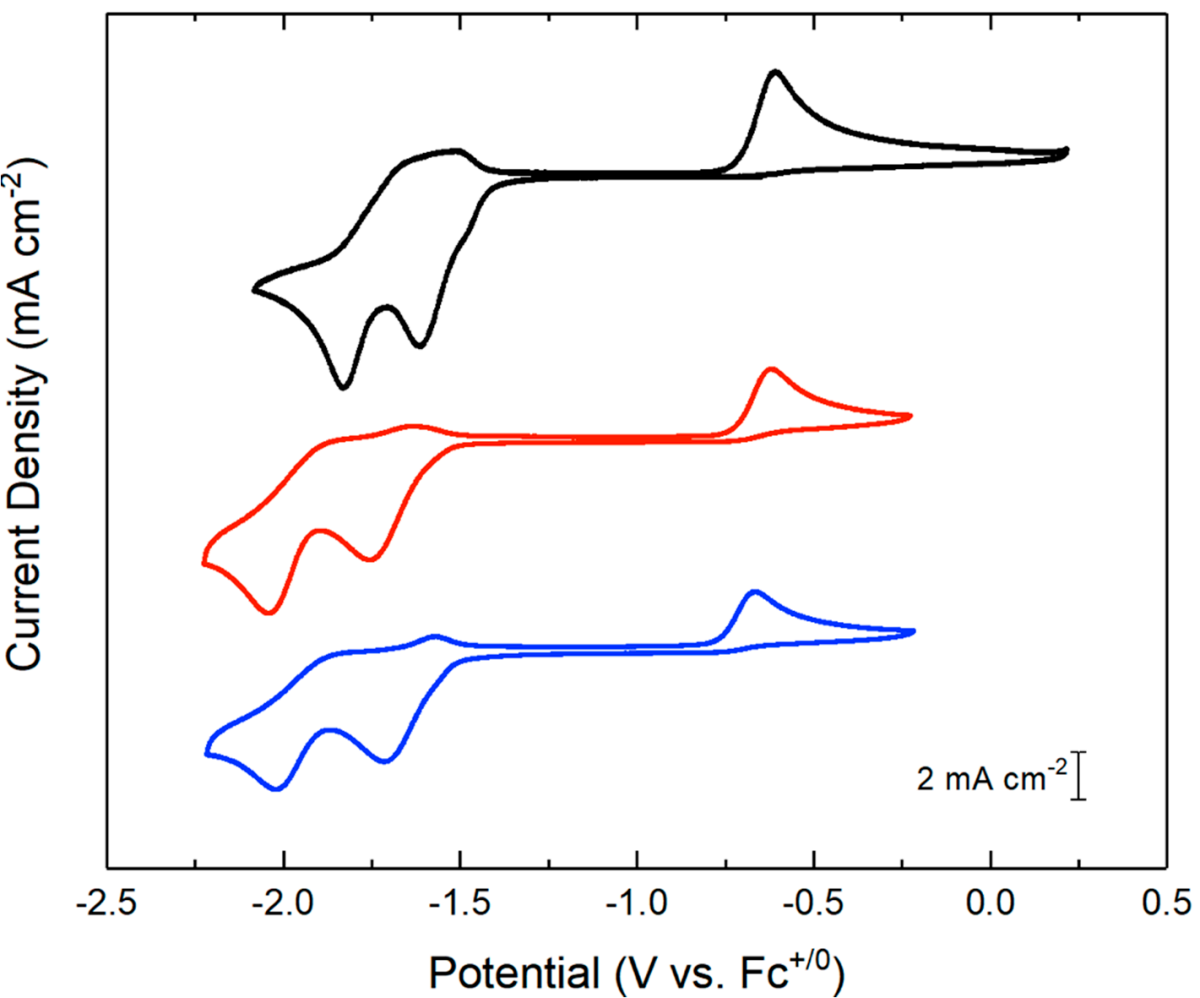
Cyclic voltammogram of complexes **1** (black), **2** (red), and **3** (blue) in MeCN solution with 0.1 M TBAPF_6_ electrolyte (WE: HOPG, Psuedo Ref: Ag^+/0^, CE: Pt, internal Ref: Fc^+/0^).

The electrochemical behavior of **1** was previously established by Deronzier, Chardon-Noblat, and co-workers [4]. We have confirmed their findings here for comparison purposes (see Figure 6); scanning cathodically with **1** in solution, we observe two irreversible reductions with cathodic peak potentials (*E*_p,c_) at −1.61 V and −1.83 V, followed by an oxidation at a more positive potential (*E*_p,a_ = −0.61 V). Based upon extensive mechanistic work from prior studies, the first reduction of **1** is associated with formation of a 19 e^−^ complex (an electron transfer or E process) which is coupled to the loss of bromide that generates a 17 e^−^ species (a chemical reaction or C process). This 17 e^−^ complex then dimerizes with itself (C process), forming [Mn(CO)_3_(bpy)]_2_ in an overall ECC-type process. [Mn(CO)_3_(bpy)]_2_ itself can then undergo reduction at the more negative potential, breaking the dimer to form [Mn(CO)_3_(bpy)]^−^ in an EC-type process. Finally, scanning anodically, oxidation of [Mn(CO)_3_(bpy)]_2_ can regenerate the starting material **1**. 

The cyclic voltammetric profiles of **2** and **3** are very similar to that associated with **1** (See Figure 6). Scanning cathodically with **2** or **3**, two irreversible reductions and followed by an oxidation at more positive potentials during the paired anodic sweep (for **2**, *E*^1^_p,c_ = −1.75 V, *E*^2^_p,c_ = −2.04 V, *E*_p,a_ = −0.62 V; for **3**, *E*^1^_p,c_ = −1.71 V, *E*^2^_p,c_ = −2.02 V, *E*_p,a_ = −0.67 V). Qualitatively, these results suggest that the irreversible reductions corresponding to the ECC and EC processes exhibited by **1** also occur with **2** and **3**. Notably, however, the reduction events associated with **2** and **3** appear significantly broader than those associated with **1**, suggesting that heterogeneous electron transfer is slower with the diazafluorene derivatives. Furthermore, as *E*^1^_p,c_ and *E*^2^_p,c_ are both more negative for **2** than **3**, we anticipate that electron transfer kinetics dominate the potentials measured for these reductions; Me_2_daf might have been expected to engender a more negative reduction potential for **3** over the case of daf in **2**, but the opposite is in fact observed here; this may be attributable to the influence of the disparate electron-transfer kinetics, which push the reduction potential (*E*^1^_p,c_) of **2** to a more negative potential than **3**, contrary to the thermodynamic trend that would be predicted on the basis of the inductive effect of the methyl groups of Me_2_daf.

Encouraged by the similar cyclic voltammetry (CV) behavior displayed by **1**, **2**, and **3**, we also tested the new compounds for activity toward CO_2_ reduction (see Figures S34–S39) since the known **1** has been demonstrated to be a robust catalyst for CO generation from CO_2_ [4]. For this testing, water was added as a proton source (similar to the prior work with **1** described in reference [4]) and CO_2_ was sparged through the working solution and electrochemical cell to fully saturate the atmosphere and solution. Voltammograms collected immediately following these additions reveal enhancements in the current flowing at both the first and second irreversible reductions associated with **2** and **3**. The observed current enhancement suggests that significant reduction-induced reactivity is taking place at the electrode surface. Notably, the overall catalytic enhancement encountered with **2** is significantly greater than that with **3**, suggesting a unique role of the acidic protons on the methylene bridge of daf in promoting reactivity. 

However, controlled potential electrolysis (CPE) coupled to product detection does not suggest effective catalytic reduction of CO_2_ is taking place with **2** and **3**. Results from controlled potential electrolyses at −2.05 V vs. Fc^+/0^ for 90 min (see Figures S40 and S41) in a custom two-compartment electrochemical cell do show that experiments with **2** and **3** produce less H_2_ and more CO during the 90 min electrolysis (see Table S1). However, **2** and **3** give low Faradic efficiencies (27% and 34%) and sub-stoichiometric yields (turnovers of 0.82 and 0.62, respectively) of CO, on the basis of total charge passed and initial loading of **2** or **3**, respectively. As analysis of working solutions with NMR spectroscopy following electrolysis did not reveal the presence of alternative products, including formate, we conclude that electrochemical reduction of **2** or **3** in the presence of H_2_O and CO_2_ leads primarily to decomposition. 

On a final note, we wish to note that the chronoamperogram associated with electrolysis of **2** is considerably different than that of **3**. The current passed as a function of time widely fluctuated during the course of the electrolysis (see Figure S40). In particular, the initial current is rather large but becomes attenuated over the course of the experiment, suggesting undesirable chemical reactivity may be taking place with the daf ligand. These results suggest that further work is needed to reveal the precise role of the acidic methylene protons in the daf framework during conditions of redox catalysis, like those explored here. It should also be noted that both metal- and bpy-centered reductions have been implicated in effective catalysis of CO_2_ reduction with **1** [47]. As incoming CO_2_ might therefore be required to interact with both the metal and the ligand, the enhanced steric profile of Me_2_daf ligand in **3** could negatively impact the approach of CO_2_ and deactivate the catalyst. Consequently, our future work will include a focus on revealing the influence of the functionalization pattern of the 9-position of daf on redox chemistry and catalysis.

## 3. Conclusions

We have described the synthesis, characterization, and electrochemical properties of the new daf- or Me_2_daf-supported complexes **2**, **3**, and **5** and compared the properties of these compounds to their bpy-supported analogues **1** and **4**. When daf and Me_2_daf are bound to Mn or Ru centers, we observe characteristic spectra that confirm the formation and symmetry of the desired complexes. In particular, comparisons of bond lengths and geometric parameters confirm that daf and Me_2_daf enforce wider chelate angles and offer weaker σ-donation than bpy. Electrochemical studies of **5** reveal that Me_2_daf is a non-innocent redox active ligand at modestly reducing potentials, and related electrochemical work with **2** and **3** shows that this ligand-centered reduction behavior is also accessible in **2** and **3**, albeit with apparently slower heterogeneous electron transfer kinetics that those encountered with analogous **1**. Taken together, these studies demonstrate daf and Me_2_daf could be useful for preparation of a variety of new redox-active compounds, building on the significant body of findings for the workhorse bpy and ^R^bpy ligands. 

## 4. Materials and Methods 

### 4.1. General Considerations

All manipulations were carried out in dry N_2_-filled gloveboxes (Vacuum Atmospheres Co., Hawthorne, CA, USA) or under an N_2_ atmosphere using standard Schlenk techniques unless otherwise noted. All solvents were of commercial grade and dried over activated alumina using a PPT Glass Contour (Nashua, NH, USA) solvent purification system prior to use, and were stored over molecular sieves. All chemicals were obtained from major commercial suppliers. Manganese pentacarbonyl bromide (98%, Strem Chemical Co., Newburyport, MA, USA), ruthenium chloride hydrate (Pressure Chemical Co., Pittsburgh, PA, USA), and 1,10-phenantrholine (95%, Matrix Scientific, Columbia, SC, USA) were used as received. The ligands, 4,5-diazafluorene and 9,9-dimethyldiazafluorene were prepared according to literature methods with minor modifications [27,28]. 4,5-diazafluorene can be sublimed at ca. 80 °C and 1 mTorr if pre-purification is necessary. Deuterated solvents for NMR studies were purchased from Cambridge Isotope Laboratories (Tewksbury, MA, USA); CD_3_CN was dried over molecular sieves. ^1^H-, ^13^C-, ^19^F-, and ^31^P-NMR spectra were collected on 400 or 500 MHz Bruker spectrometers (Bruker, Billerica, MA, USA) and referenced to the residual protio-solvent signal in the case of ^1^H and ^13^C [48]. Heteronuclear NMR spectra were referenced to the appropriate external standard following the recommended scale based on ratios of absolute frequencies (Ξ) [49,50]. ^19^F NMR spectra are reported relative to CCl_3_F, and ^31^P NMR spectra are reported relative to H_3_PO_4_. Chemical shifts (δ) are reported in units of ppm and coupling constants (*J*) are reported in Hz. Elemental analyses were performed by Midwest Microlab, Inc. (Indianapolis, IN, USA).

Electronic absorption spectra were collected with an Ocean Optics Flame spectrometer equipped with a DH-Mini light source (Ocean Optics, Largo, FL, USA).

IR spectra were collected using a Shimadzu IRSpirit Fourier transform infrared spectrometer in transmission mode using a 0.1 cm liquid IR cell with KBr windows. 

### 4.2. X-Ray Crystallography

Single-crystal diffraction data were collected with a Bruker APEX-II CCD diffractometer. The Cambridge Crystallographic Data Centre (CCDC) entries 1977431, 1994285, 1982214, and 2013030 contain the supplementary crystallographic data for complexes **2**, **3**, and **5** (v74e and q36k), respectively. These data can be obtained free of charge via www.ccdc.cam.ac.uk/data_request/cif, or by emailing data_request@ccdc.cam.ac.uk, or by contacting The Cambridge Crystallographic Data Centre, 12, Union Road, Cambridge CB2 1EZ, UK; fax: +44 1223 336033.

### 4.3. Electrochemistry

Electrochemical experiments were performed in a N_2_-filled glovebox, or outside of the box in an argon-flushed electrochemical cell. Dry, degassed MeCN and 0.1 M tetra(*n*-*butyl*)ammonium hexafluorophosphate ([^n^Bu_4_N]^+^[PF_6_]^−^ (Sigma-Aldrich, electrochemical grade) were used as the solvent and supporting electrolyte. Measurements were carried out with Reference 600+ Potentiostat/Galvanostat (Gamry Instruments, Warminster, PA, USA), or an electrochemical analyzer potentiostat (CH Instruments), using a standard three-electrode configuration. For CV experiments: the working electrode was the basal plane of highly oriented pyrolytic graphite (HOPG) (GraphiteStore.com, Buffalo Grove, IL, USA; surface area: 0.09 cm^2^), the counter electrode was a platinum wire (Kurt J. Lesker, Jefferson Hills, PA, USA; 99.99%, 0.5 mm diameter), and a silver wire immersed in electrolyte solution served as a pseudo-reference electrode (CH instruments). The reference was separated from the working solution by a Vycor frit (Bioanalytical Systems, Inc., West Lafayette, IN, USA). For CV acid addition experiments: the working electrode was the basal plane of HOPG (surface area: 0.09 cm^2^), the counter and reference electrodes were platinum wires (99.99%, 0.5 mm diameter). Ferrocene (Sigma-Aldrich, St. Louis, MO, USA; twice-sublimed) was added to the electrolyte solution at the end of each experiment; the midpoint potential of the ferrocenium/ferrocene couple (denoted as Fc^+/0^) was used as an external standard for comparison of the recorded potentials. Concentrations of the analytes for cyclic voltammetry were typically 1 mM. Experiments were typically conducted by first scanning cathodically, then anodically on the return sweep. 

Bulk electrolysis experiments were performed in a custom two-chamber electrochemical cell equipped with connections to achieve gas-tight operation. The working electrode was the basal plane of HOPG (Graphitestore.com, Buffalo Grove, IL, USA; surface area: 10 cm^2^), the counter electrode was a platinum wire (99.99%, 0.5 mm diameter), and a silver wire immersed in electrolyte solution served as a pseudo-reference electrode. The volume of solution held by the cell in total was 60 mL, with about 105 mL of total head-space volume. 

### 4.4. Gas Chromatography

Gas chromatography were collected with a Shimadzu GC-2014 Custom-GC gas chromatograph with a thermal conductivity detector and dual flame-ionization detectors. A custom set of eight columns and timed valves enable quantitative analysis of the following gases: hydrogen, nitrogen, oxygen, carbon dioxide, carbon monoxide, methane, ethane, ethylene, and ethyne. Argon serves as the carrier gas. The instrument was calibrated with a standard checkout gas mixture (Agilent 5190-0519, Santa Clara, CA, USA) prior to experimental runs to obtain quantitative data for CO and other gases. Calibration curves over a range of 100–35,000 ppm were constructed with prepared mixture of CO and N_2_ to enable CO quantification. 

### 4.5. Preparation of Mn(CO)_3_(4,5-diazafluorene)Br (***2***)

In the dark, to a 50 mL Schlenk flask equipped with a stir bar, was added 4,5-diazafluorene (0.0644 g, 0.383 mmol) in 50 mL of diethyl ether. Then Mn(CO)_5_Br (0.0998 g, 0.363 mmol) was added and the reaction was brought to reflux. The reaction was monitored by ^1^H NMR until consumption of the starting material was observed to be complete, after approximately 3 hours. Once the reaction had reached completion, the Schlenk flask was placed into a refrigerator at −20 °C for 30 min. The resulting solid was then filtered off with a fritted glass funnel and washed with cold pentane to afford the title compound as a yellow solid. Yield: 0.088 g (62%). ^1^H-NMR (CD_3_CN, 500 MHz) δ 8.85 (d, ^3^*J*_H–H_ = 5.3 Hz, 2H), 8.14 (d, ^3^*J*_H–H_ = 7.6 Hz, 2H), 7.61–7.58 (dd, ^3^*J*_H–H_ = 7.6 Hz, ^4^*J*_H–H_ = 5.6 Hz, 2H), 4.29 (d, ^2^*J*_H–H_ = 22.6 Hz, 2H) ppm. ^13^C{^1^H} NMR (176 MHz, CD3CN): δ 162.3, 151.3, 137.7, 136.5, 126.9, 37.6 ppm. ^13^C{^1^H}-DEPT-135 NMR δ 151.2, 136.4, 126.9, 37.5 ppm. Electronic absorption spectrum (MeCN): 230 (16,000), 297 (9970), 301 (9910), 311 (10,100), 320 (10,400), 327 (10,700), 410 nm (2200 M^−1^ cm^−1^). IR (THF): ν_C=O_ 2026 (m) (A’), ν_C=O_ 1938 (m) (A’’), and ν_C=O_ 1917 (m) (A’) cm^−1^. ESI-MS (positive) *m*/*z*: 348.0 (98%) (**1**–Br^−^+NCMe), 349.0 (18%), 350.0 (2%); 306.9 (29%) (**1**–Br^−^), 307.9 (5%), 308.9 (0.5%); 305.0 (96%) (**1**–Br^−^–3CO+2NCMe), 306.0 (18%); 264.0 (45%) (**1**–Br^−^–3CO+NCMe), 265.0 (7%); 223.0 (100%) (**1**–Br^−^–3CO), 224.0 (13%). Anal. Calcd. for MnC_14_H_8_BrN_2_O_3_: C, 43.44; H, 2.08; N, 7.24. Found: C, 43.38; H, 2.08; N, 7.14. 

### 4.6. Preparation of Mn(CO)_3_(9,9’-dimethyl-4,5-diazafluorene)Br (***3***)

In the dark, to a Schlenk flask equipped with a stir bar was added 9,9’-dimethyl-4,5-diazafluorene (0.0749 g, 0.364 mmol) and 50 mL of diethyl ether. Then Mn(CO)_5_Br (0.1000g, 0.382 mmol) was added and the reaction was brought to reflux. The reaction was monitored by ^1^H NMR until consumption of the starting material was observed to be complete, after approximately 3 hours. Once the reaction had reached completion the Schlenk flask was placed into a −20 °C refrigerator for 30 minutes. The resulting solid was then filtered off with a fritted glass funnel and washed with cold Et_2_O to afford the title compound as a yellow solid. Yield: 0.1098 g (73%). ^1^H-NMR (CD_3_CN, 500 MHz) δ 8.82 (d, ^3^*J*_H–H_ = 5.3 Hz, 2H), 8.10 (d, ^3^*J*_H–H_ = 7.7 Hz, 2H), 7.59 (dd, ^3^*J*_H–H_ = 7.7 Hz, ^4^*J*_H–H_ = 5.3 Hz, 2H), 1.66 (s, 3H), 1.58 (s, 3H) ppm. ^13^C{^1^H} NMR (176 MHz, CD_3_CN): δ 160.3, 151.5, 147.2, 134.0, 127.5, 52.1, 25.3, 24.4 ppm. Electronic absorption spectrum (MeCN): 236 (15,000), 301 (11,000), 306 (11,000), 316 (11,600), 324 (12,000), 332 (13,000), 411 nm (3300 M^−1^ cm^−1^). IR (THF): ν_C=O_ 2026 (m) (A’), ν_C=O_ 1938 (m) (A’’), and ν_C=O_ 1915 (m) (A’) cm^−1^. ESI-MS (positive) *m*/*z*: 251.0 (100%) (**1**–Br^−^–3CO), 252.0 (15%), 253.0 (1%). Anal. Calcd. for MnC_16_H_12_BrN_2_O_3_: C, 46.29; H, 2.91; N, 6.75. Found: C, 46.35; H, 3.03; N, 6.97.

### 4.7. Preparation of [Tris(9,9’-dimethyl-4,5-diazafluorene)Ruthenium](PF_6_)_2_ (***5***)

In the dark, to a three-neck round bottom flask equipped with a stir bar was added 9,9’-dimethyl-4,5-diazafluorene (0.1000 g, 0.509 mmol), RuCl_3_ × H_2_O (0.0266 g, 0.128 mmol), and Zn^0^ powder (0.0420 g, 0.642 mmol). A 2:1 ethanol:water mixture was used as a solvent to suspend the material, the reaction mixture was brought to reflux, and was allowed to stir overnight. The resulting bright-orange solution was then filtered into a flask containing ammonium hexafluorophosphate (0.0438 g, 0.269 mmol), which resulted in immediate precipitation of the desired product. The precipitate was filtered, and then washed progressively with cold water and diethyl ether. The desired complex was purified by recrystallization from boiling methanol to afford an orange solid. Yield (0.0210 g, 17%). ^1^H-NMR (CD_3_CN, 400 MHz) δ 8.06 (dd, ^3^*J*_H–H_ = 7.8 Hz, ^4^*J*_H–H_ = 0.9 Hz, 6H), 7.81 (dd, ^3^*J*_H–H_ = 5.5 Hz, ^4^*J*_H–H_ = 0.9 Hz, 6H), 7.44 (dd, ^3^*J*_H–H_ = 7.8 Hz, ^4^*J*_H–H_ = 5.5 Hz, 6H), 1.68 (s, 18H) ppm.^13^C{^1^H} NMR (176 MHz, CD_3_CN): δ 162.8, 152.9, 147.4, 133.4, 127.9, 53.2, 24.5 ppm. ^19^F NMR (276 MHz, CD_3_CN): δ −72.9 (d, 706.4 Hz) ppm.^31^P NMR (162 MHz, CD_3_CN): δ −144.7 (m, 706.4 Hz) ppm. Electronic absorption spectrum (MeCN): 231 (27,000), 249 (14,400), 256 (13,500), 295 (75,000), 445 nm (17,000 M^−1^ cm^−1^). Anal. Calcd. for RuC_29_H_36_N_6_F_12_P_2_: C, 47.81; H, 3.70; N, 8.58. Found: C, 47.62; H, 3.70; N, 8.30.

## Supplementary Materials

The following are available online: NMR spectra, IR spectra, electronic absorption spectra, electrochemical, gas chromatography data, and crystallographic details (PDF); cartesian coordinates (XYZ).
