# Supplementary material for: 4,5-Diazafluorene and 9,9’-Dimethyl-4,5-Diazafluorene as Ligands Supporting Redox-Active Mn and Ru Complexes
Source: Molecules. 2020 Jul 13;25(14):3189. doi: 10.3390/molecules25143189 (PMC7396985; doi:10.3390/molecules25143189)
Supplement: Supplementary file 1 [file molecules-25-03189-s001.pdf]

Supporting Information for:

4,5-Diazafluorene and 9,9′-Dimethyl-4,5-Diazafluorene as Ligands Supporting Redox-Active Mn and Ru Complexes

*Wade C. Henke, Julie A. Hopkins, Micah L. Anderson, Jonah P. Stiel, Victor W. Day, and James D. Blakemore**

Department of Chemistry, University of Kansas
1567 Irving Hill Road, Lawrence, KS 66045

* To whom correspondence should be addressed: E-mail: blakemore@ku.edu
phone: +1 (785) 864-3019

Contents

Chart S1: Ligands and Complexes of Interest S3

NMR Spectra
Figure S1: ^{1}H-NMR Spectrum of **2** S4
Figure S2: $^{13}C\{^{1}H\}$-NMR Spectrum of **2** S4
Figure S3: $^{13}C\{^{1}H\}$ DEPT-NMR Spectrum of **2** S4
Figure S4: ^{1}H-NMR Spectrum of **3** S5
Figure S5: $^{13}C\{^{1}H\}$-NMR Spectrum of **3** S5
Figure S6: ^{1}H-NMR Spectrum of **5** S6
Figure S7: $^{13}C\{^{1}H\}$-NMR Spectrum of **5** S6
Figure S8: ^{19}F-NMR Spectrum of **5** S6
Figure S9: ^{31}P-NMR Spectrum of **5** S7
Figure S10: ^{1}H-^{13}C HSQC Spectrum of **2** S8
Figure S11: ^{1}H-^{13}C HSQC Spectrum of **3** S9

IR Spectra
Figure S12: Stacked IR Spectrum of **1**, **2**, and **3** S10

Mass Spectra
Figure S13: Full ESI Mass Spectrum of **2** S11
Figure S14: Zoomed in ESI Mass Spectrum of **2** S11
Figure S15: Full ESI Mass Spectrum of **3** S12
Figure S16: Zoomed in ESI Mass Spectrum of **3** S12

UV-Vis Spectra
Figure S17: Stacked UV-Vis Spectrum of **1**, **2**, and **3** in MeCN solution S13
Figure S18: UV-Vis Spectrum of **1** in MeCN S14
Figure S19: Absorbance vs Concentration of **1** in MeCN S14
Figure S20: UV-Vis Spectrum of **2** in MeCN S15

Figure S21: Absorbance vs Concentration of **2** in MeCN S15
Figure S22: UV-Vis Spectrum of **3** in MeCN S16
Figure S23: Absorbance vs Concentration of **3** in MeCN S16
Figure S24: UV-Vis Spectrum of **5** in MeCN S17
Figure S25: Absorbance vs Concentration of **5** in MeCN S17

Electrochemistry

Figure S26: Stacked Cyclic Voltammogram of **1, 2,** and **3** S18
Figure S27: Cyclic Voltammogram of **2** S18
Figure S28: Cyclic Voltammogram of **3** S19
Figure S29: Stacked Cyclic Voltammogram of **4** and **5** S19
Figure S30: Cyclic Voltammogram of **4** S20
Figure S31: Cyclic Voltammogram of **4** with Blank S20
Figure S32: Cyclic Voltammogram of **5** S21
Figure S33: Cyclic Voltammogram of **5** with Blank S21

Electrochemistry with Acid and CO_2

Figure S34: Cyclic Voltammogram of **2** with acid only S22
Figure S35: Cyclic Voltammogram of **2** with acid and CO_2 .... S22
Figure S36: Cyclic Voltammogram of **2** with acid and CO_2 and Blank S23
Figure S37: Cyclic Voltammogram of **3** with acid only S23
Figure S38: Cyclic Voltammogram of **3** with acid and CO_2 .... S24
Figure S39: Cyclic Voltammogram of **3** with acid and CO_2 and Blank S24

Bulk Electrolysis

Figure S40: Chronoamperogram for Complexes **2** and **3** with Blank S25
Figure S41: Charge Passed as a Function of Time for Complexes **2** and **3** with Blank S25
Table S1: Bulk Electrolysis Results for complexes **2** and **3** S26

Crystallographic Information

Refinement Details S27
Table S2: Crystal and refinement data S28
Special Refinement Details for 2 .... S30
Figure S42: Full solid-state structure of **2** S30
Special Refinement Details for 3 .... S31
Figure S43: Solid-state structure of **3** S32
Figure S44: Full solid-state structure of **3** S33
Special Refinement Details for 5 (v74e) S34
Figure S45: Solid-state structure of **5 (v74e)** S35
Figure S46: Full solid-state structure of **5 (v74e)** S36
Special Refinement Details for 5 (q36k) S37
Figure S47: Solid-state structure of **5 (q36k)** S37
Figure S48: Full solid-state structure of **5 (q36k)** S38
Table S3: Selected bond lengths comparing **5 (v74e)** and **5 (q36k)** S39
Table S4: Selected bond angles comparing **5 (v74e)** and **5 (q36k)** S40
References S41

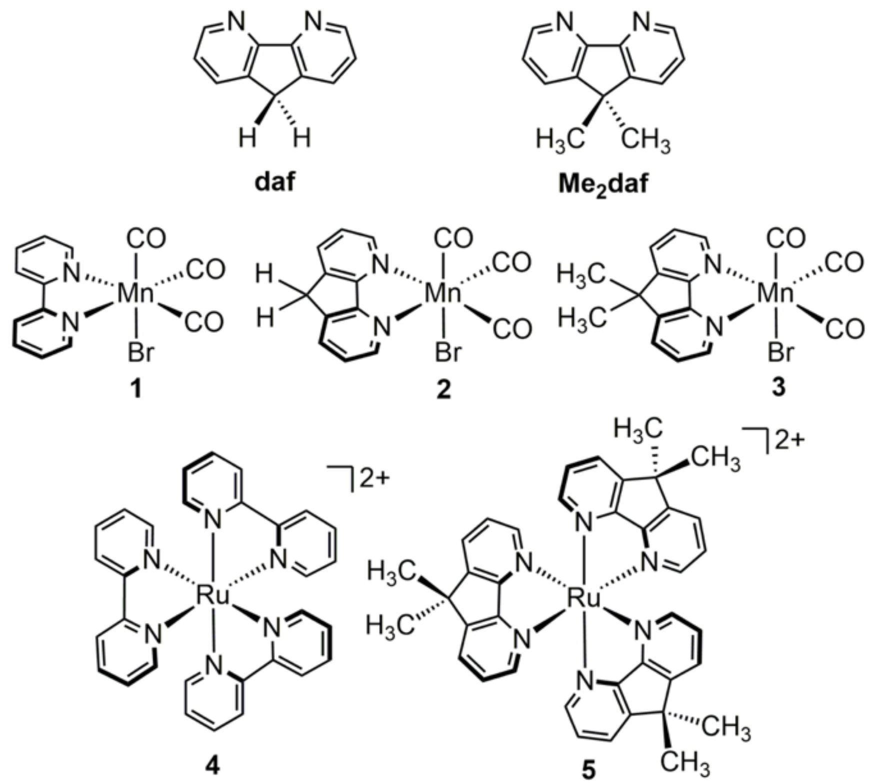

Chart S1: Ligands and complexes described in this study.

NMR Spectra

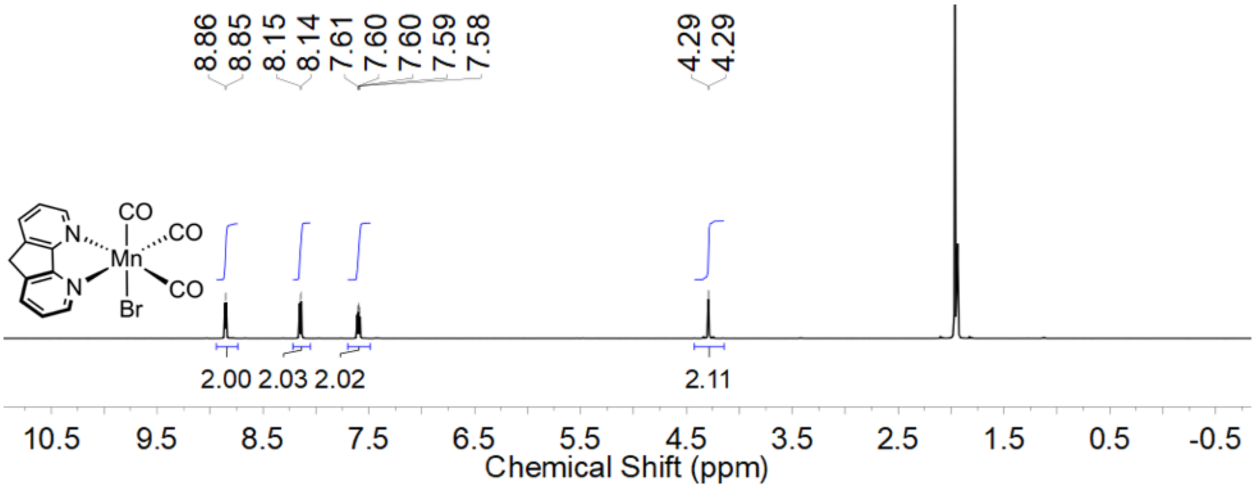

Figure S1: ^{1}H-NMR spectrum (500 MHz, CD_3CN) of **2**.

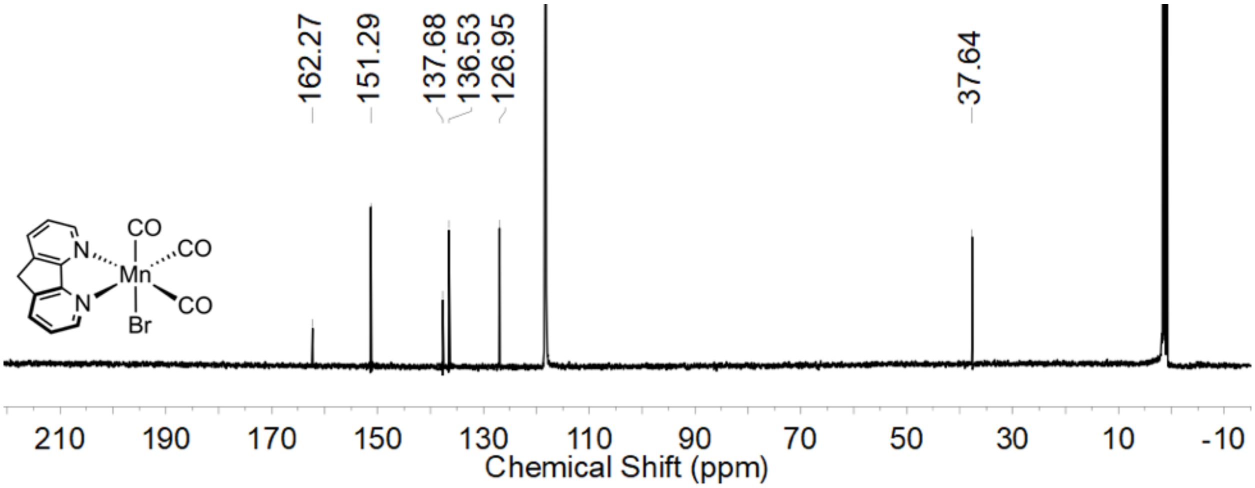

Figure S2: $^{13}C\{^{1}H\}$-NMR spectrum (126 MHz, CD_3CN) of **2**.

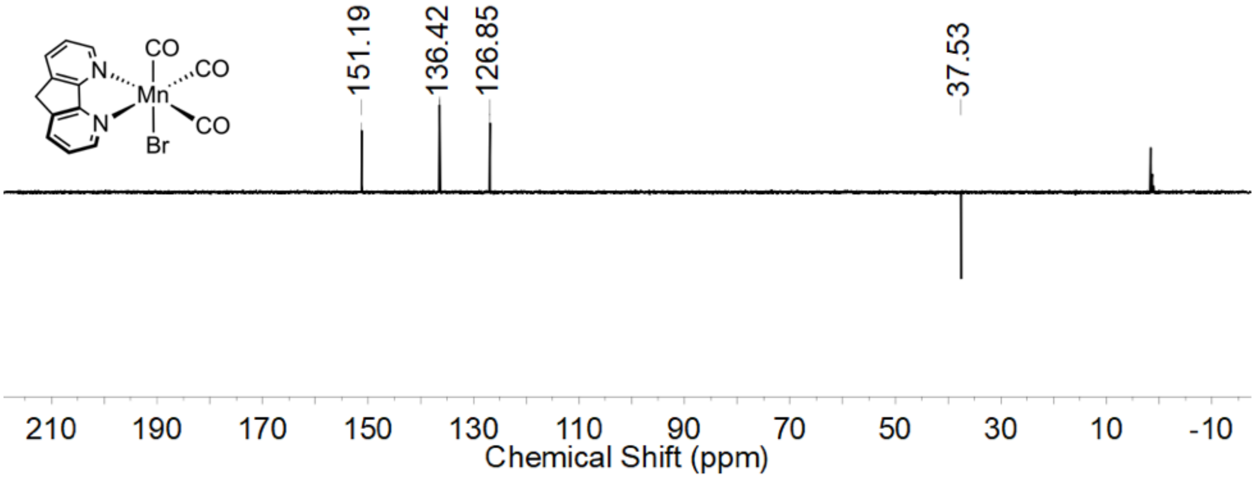

Figure S3: $^{13}C\{^{1}H\}$ DEPT-NMR spectrum (126 MHz, CD_3CN) of **2**.

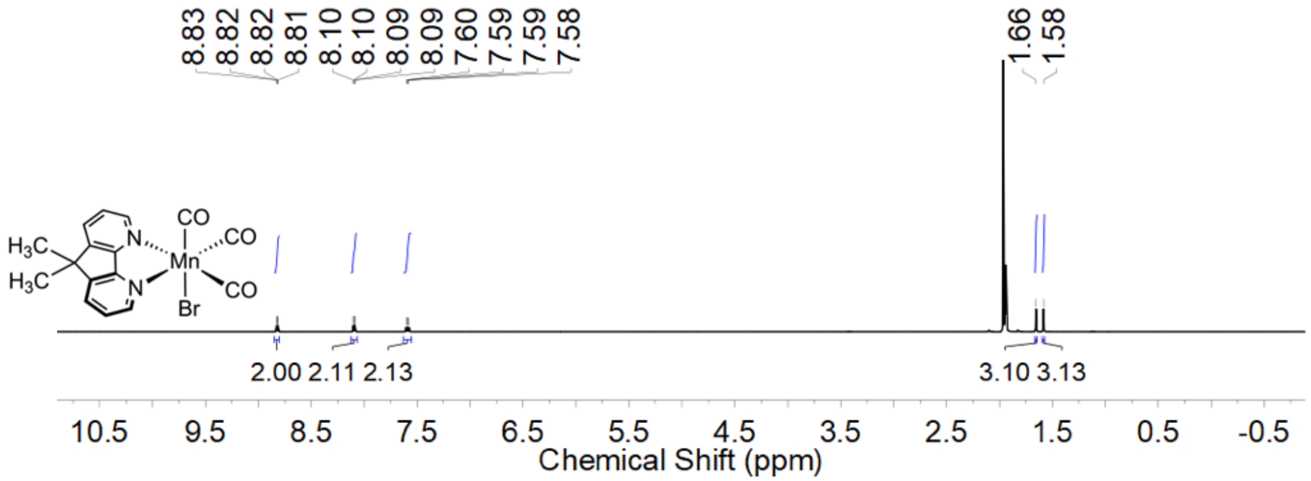

Figure S4: ^{1}H-NMR spectrum (400 MHz, CD_3CN) of **3**.

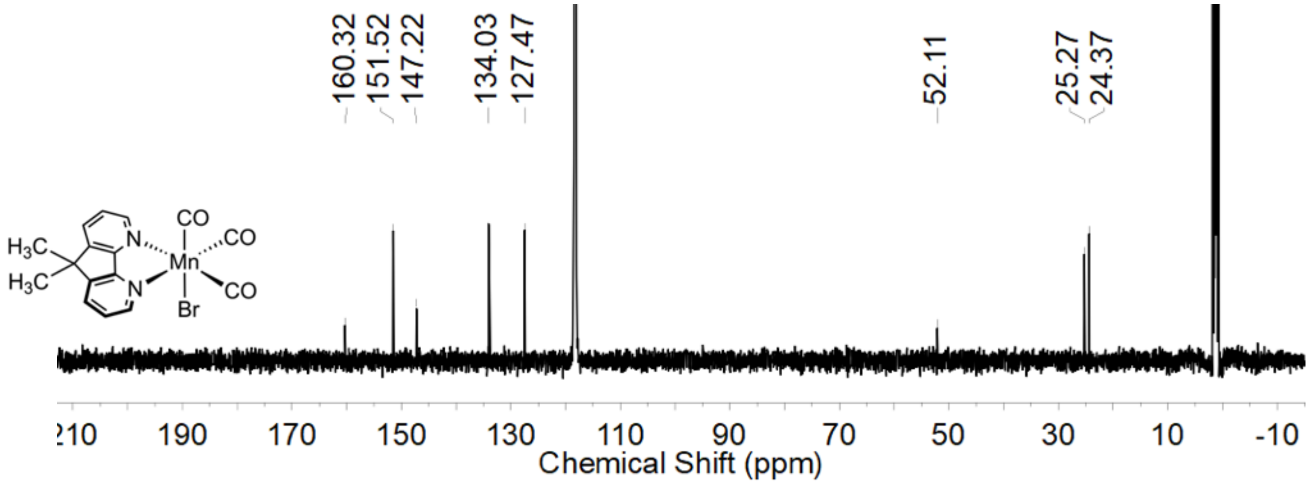

Figure S5: $^{13}C\{^{1}H\}$-NMR spectrum (126 MHz, CD_3CN) of **3**.

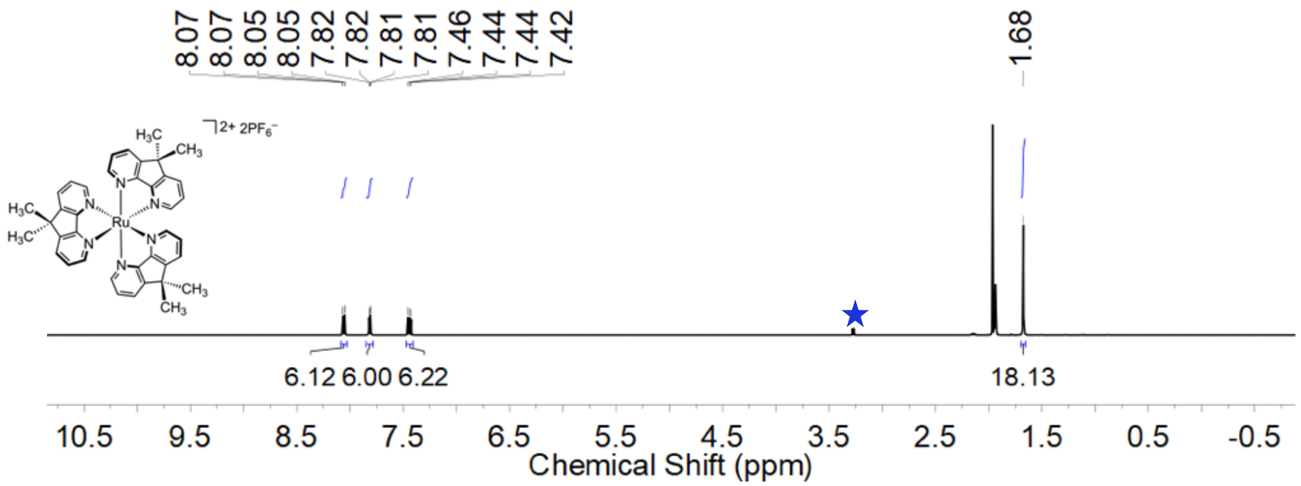

Figure S6: ^{1}H-NMR spectrum (500 MHz, CD_3CN) of **5**; The blue star (★) corresponds to adventitious methanol.

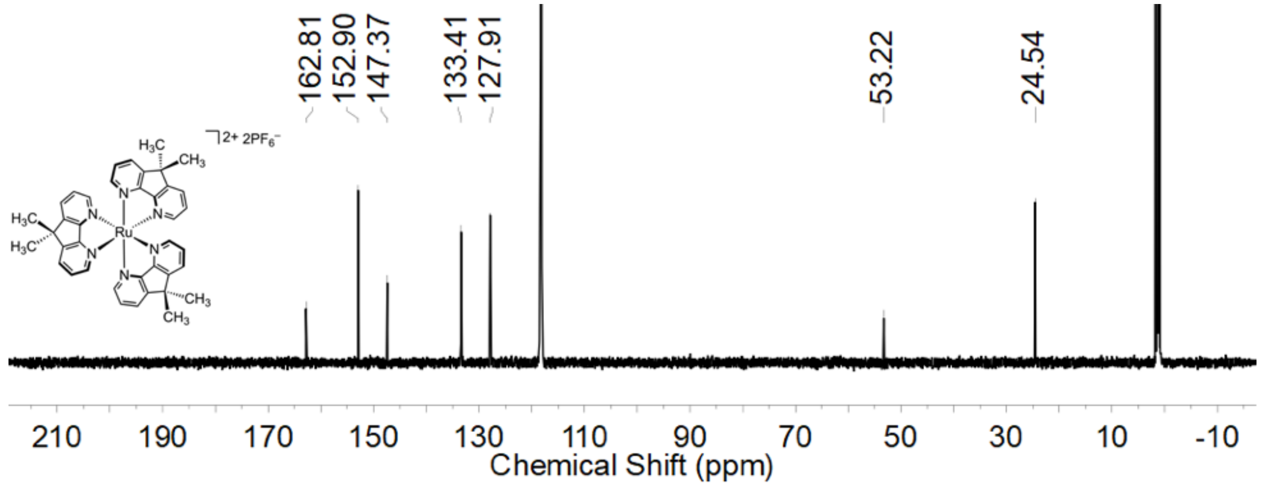

Figure S7: $^{13}C\{^{1}H\}$-NMR spectrum (126 MHz, CD_3CN) of **5**.

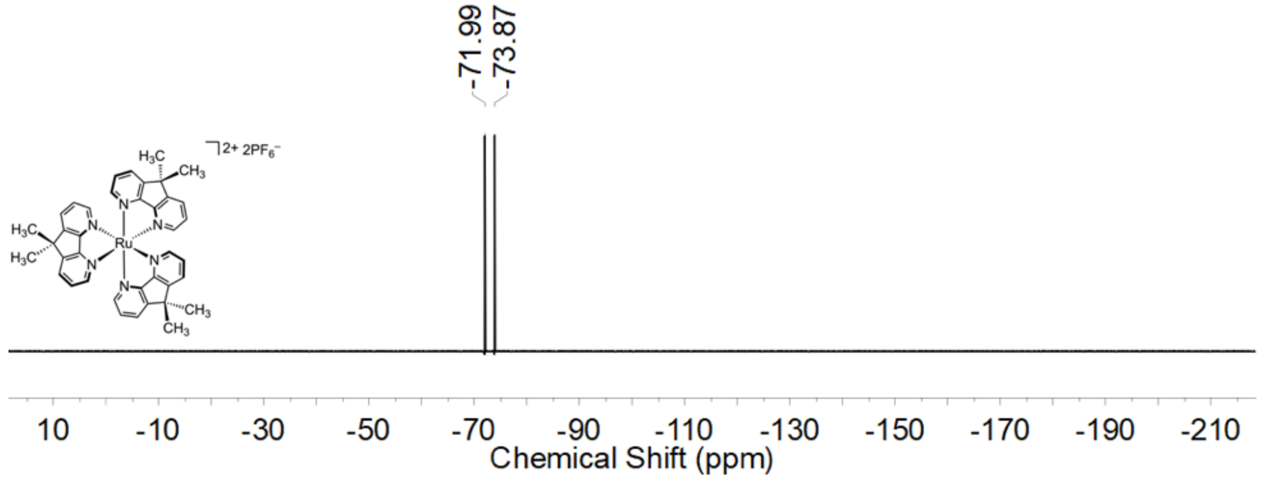

Figure S8: ^{19}F-NMR spectrum (376 MHz, CD_3CN) of **5**.

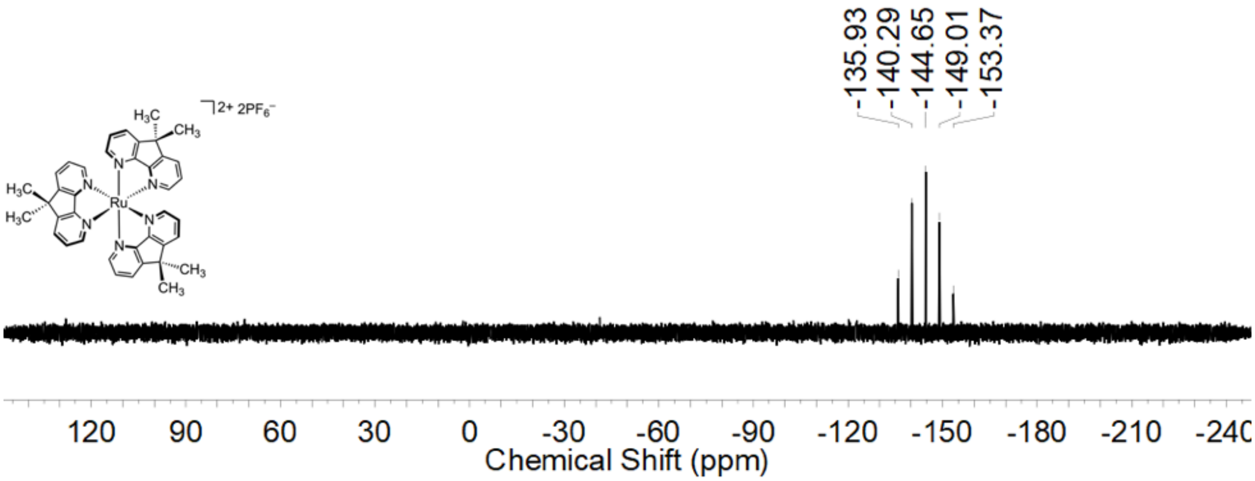

Figure S9: ^{31}P-NMR spectrum (162 MHz, CD_3CN) of **5**.

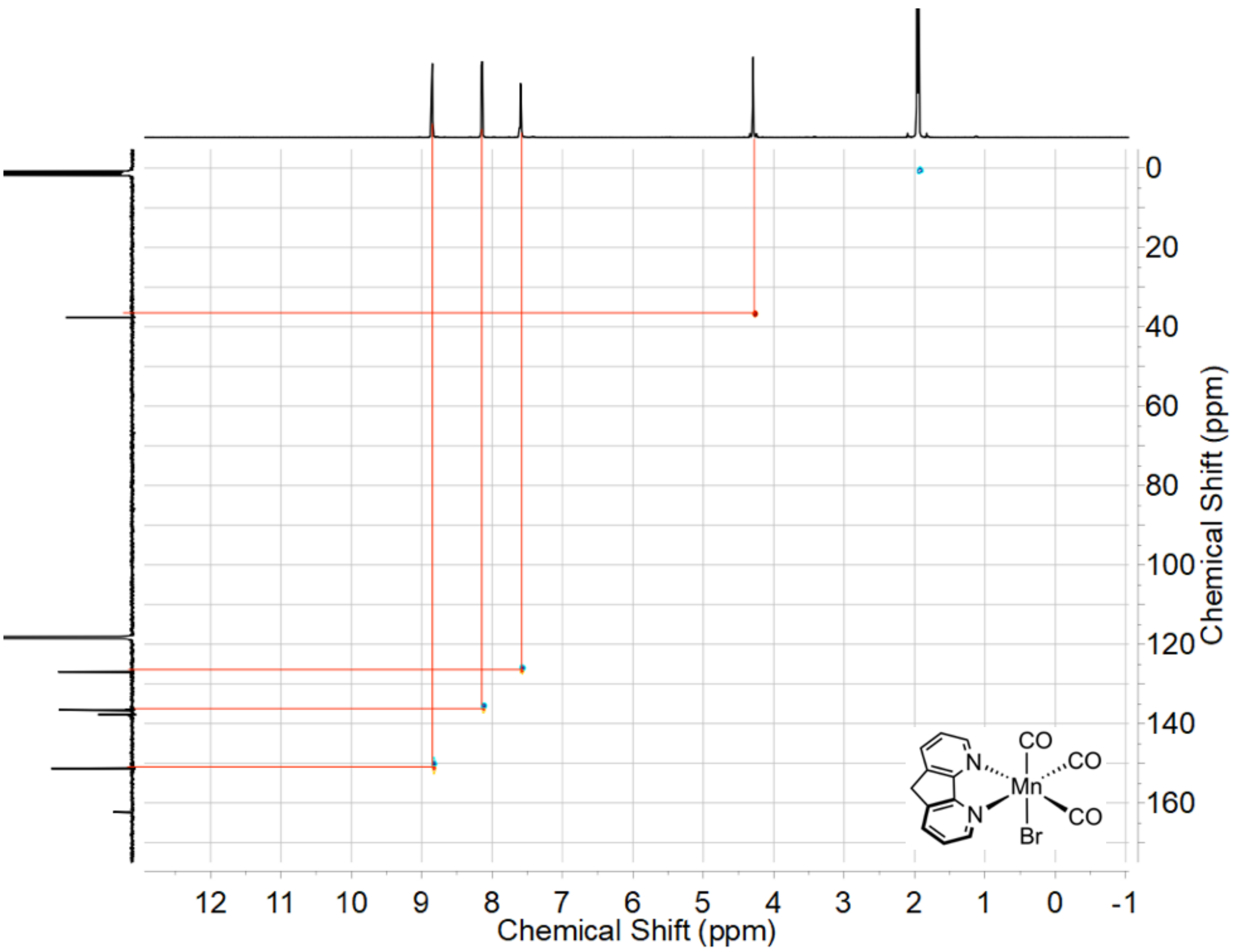

Figure S10: ^{1}H-^{13}C HSQC of complex **2** in CD_3CN depicting the methylene protons indicated by the opposite phasing (red) compared to the methyl and methine protons (blue).

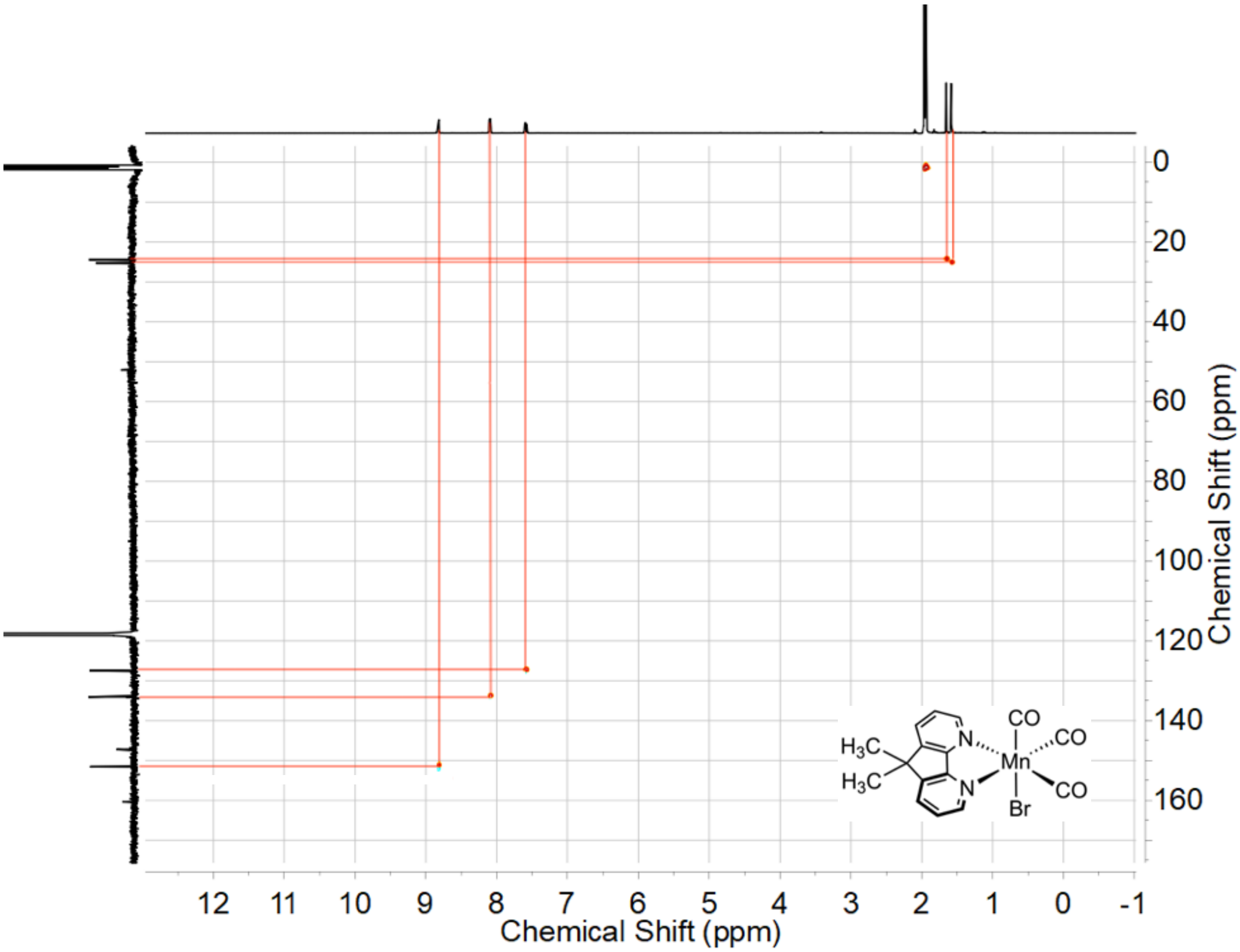

Figure S11: ^{1}H-^{13}C HSQC of complex **3** in CD_3CN depicting the diastereotopic methyl groups. Methyl and methine protons (red) and methylene protons (blue).

IR Spectra

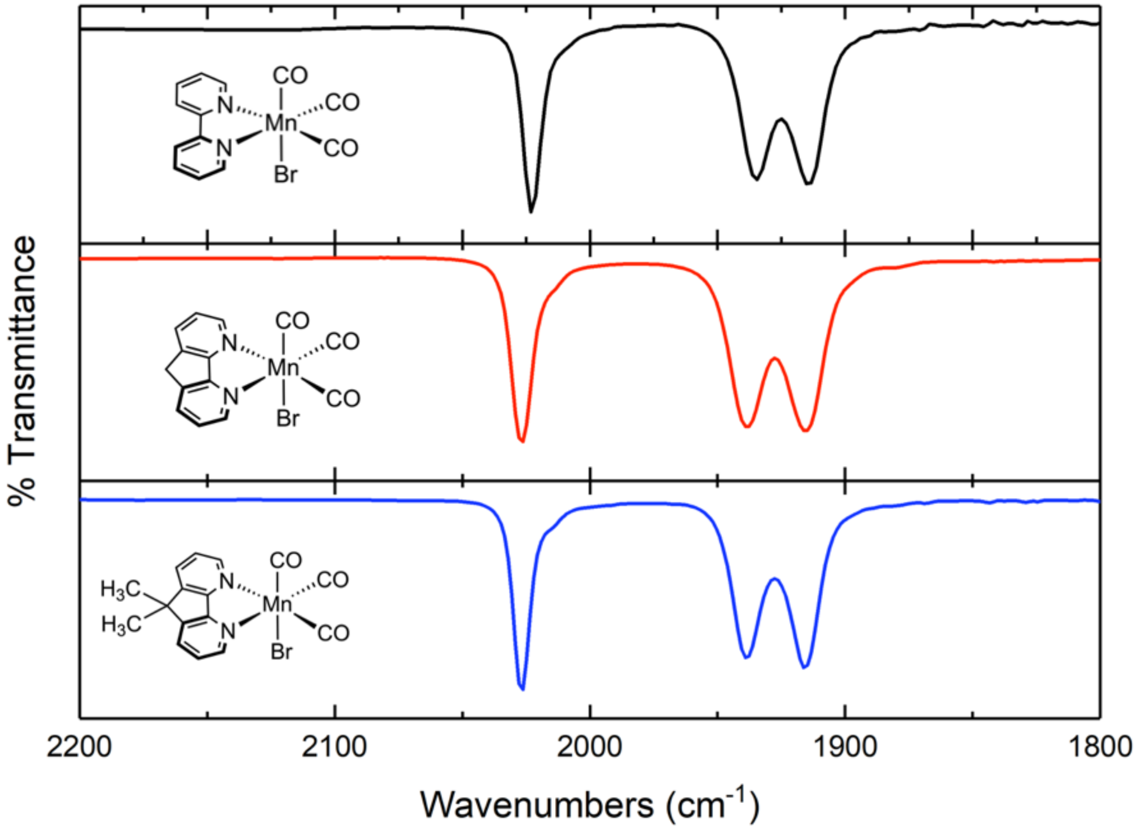

Figure S12: IR spectra of the carbonyl stretches for **1**, **2**, and **3** in THF solution; focusing on the CO stretching region. For a *fac*-tricarbonyl, C_s symmetric molecule, three CO stretches (Γ_{CO} = 2A′+A″) would be expected and are observed for complexes **1, 2,** and **3.**

Mass Spectra

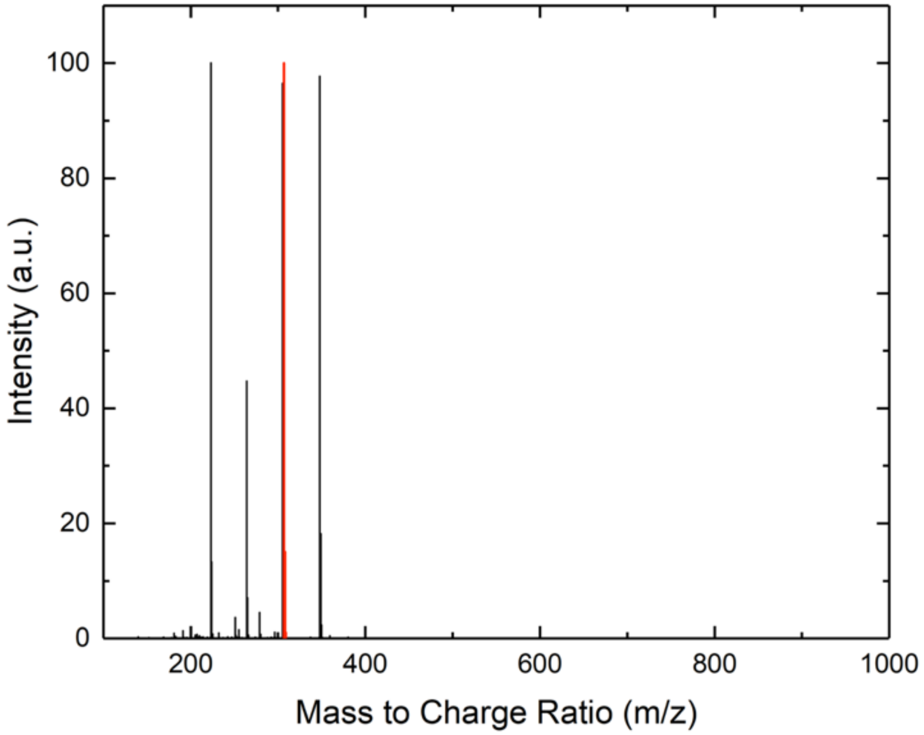

Figure S13: Full ESI-Mass spectrum of **2**. The experimental data is shown in **black** and the predicted data is shown in **red**. The other peaks in the spectrum correspond to fragments that are identified below.

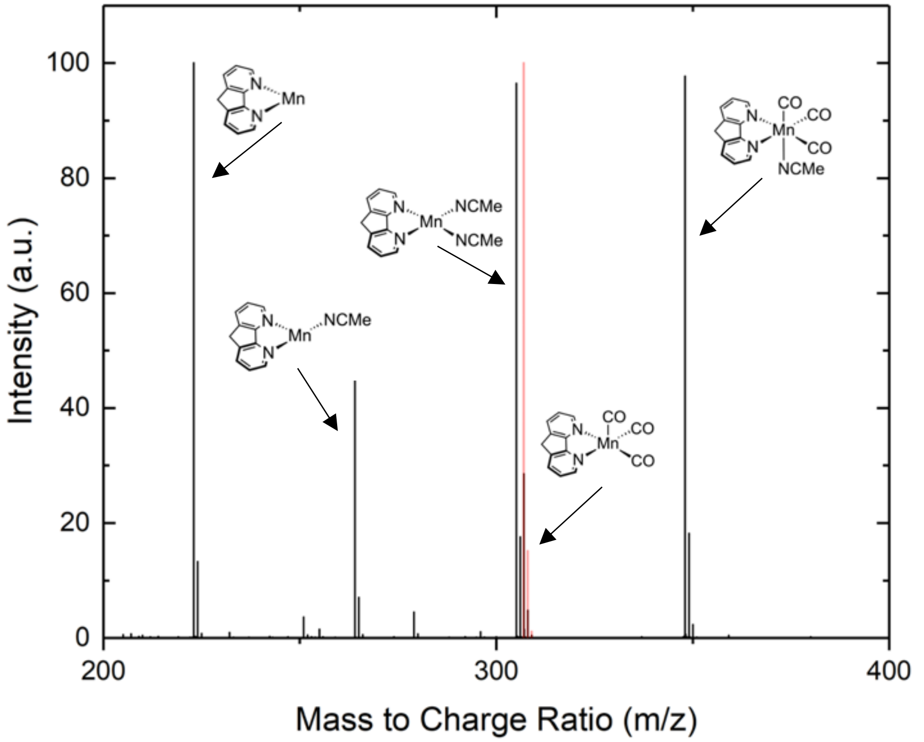

Figure S14: Zoomed in mass spectrum of **2** with fragment assignment. The experimental data is shown in **black** and the predicted data is shown in **red**.

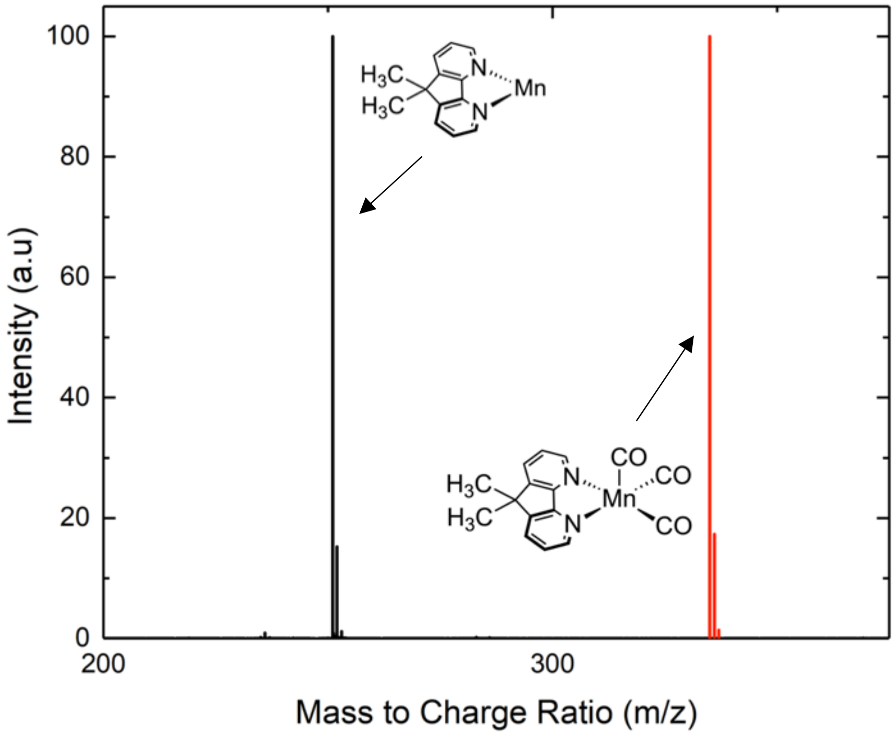

Figure S15: Full ESI-Mass spectrum of **3**. The experimental data is shown in **black** and the predicted data is shown in **red**. The expected parent peak was not present, but a corresponding fragment is identified in Figure S16.

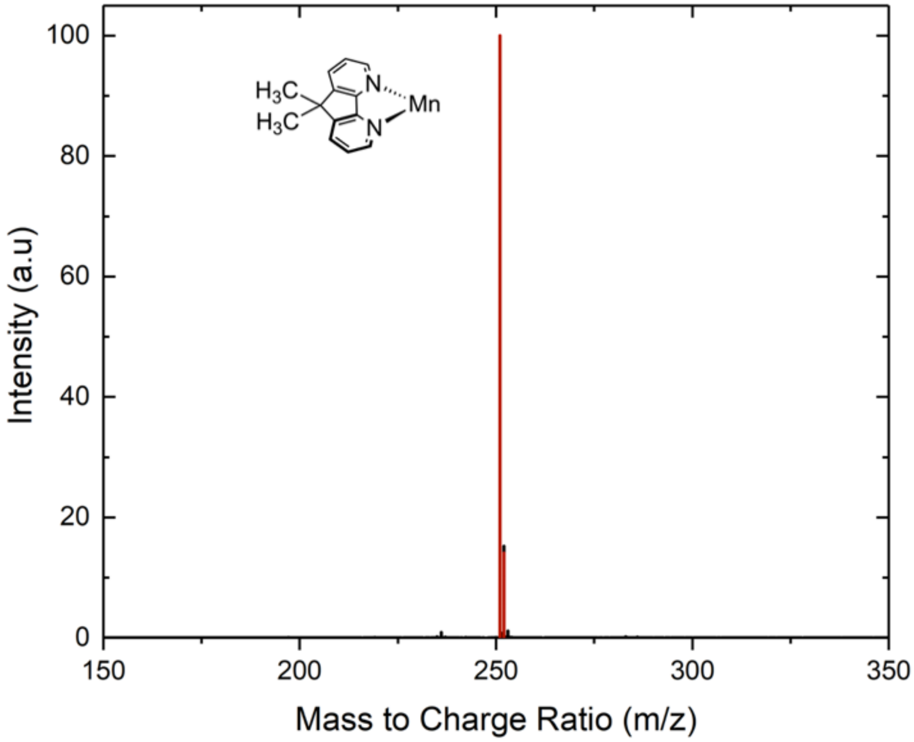

Figure S16: Zoomed in mass spectrum of **3** with fragment assignment. The experimental data is shown in **black** and the predicted data is shown in **red** for this specific fragment.

Electronic Absorption Spectra

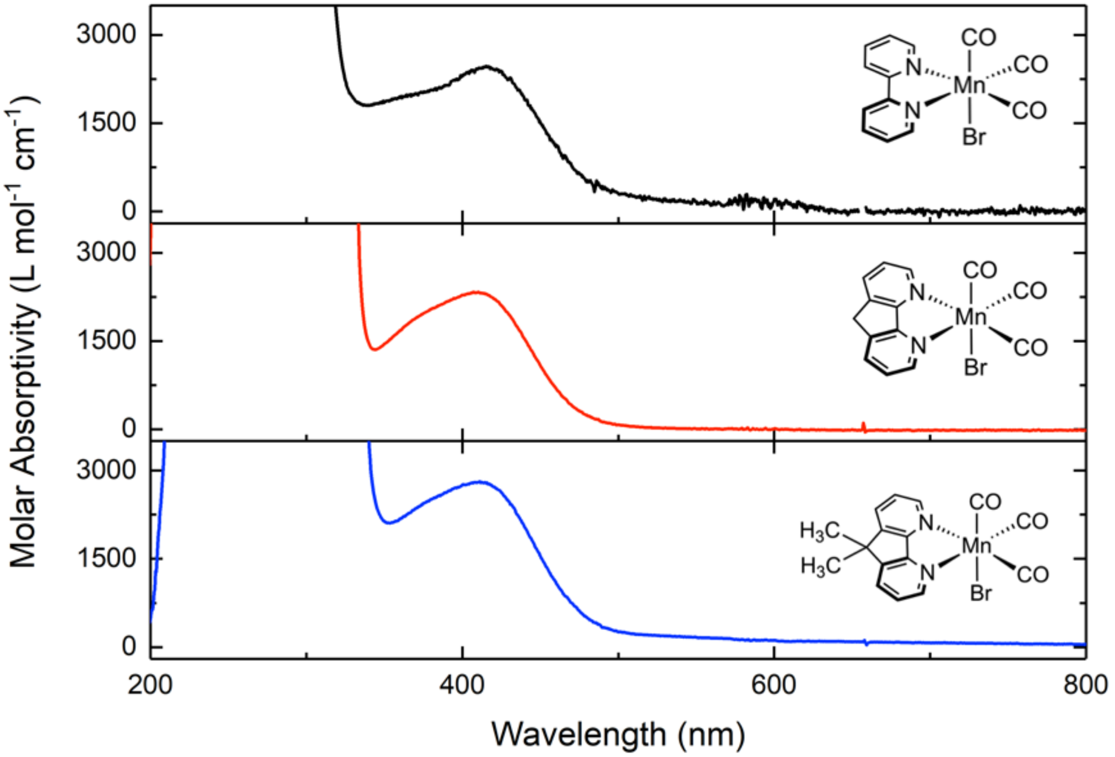

Figure S17: Stacked electronic absorption spectrum of **1**, **2**, and **3** featuring the MLCT transition in the visible region.

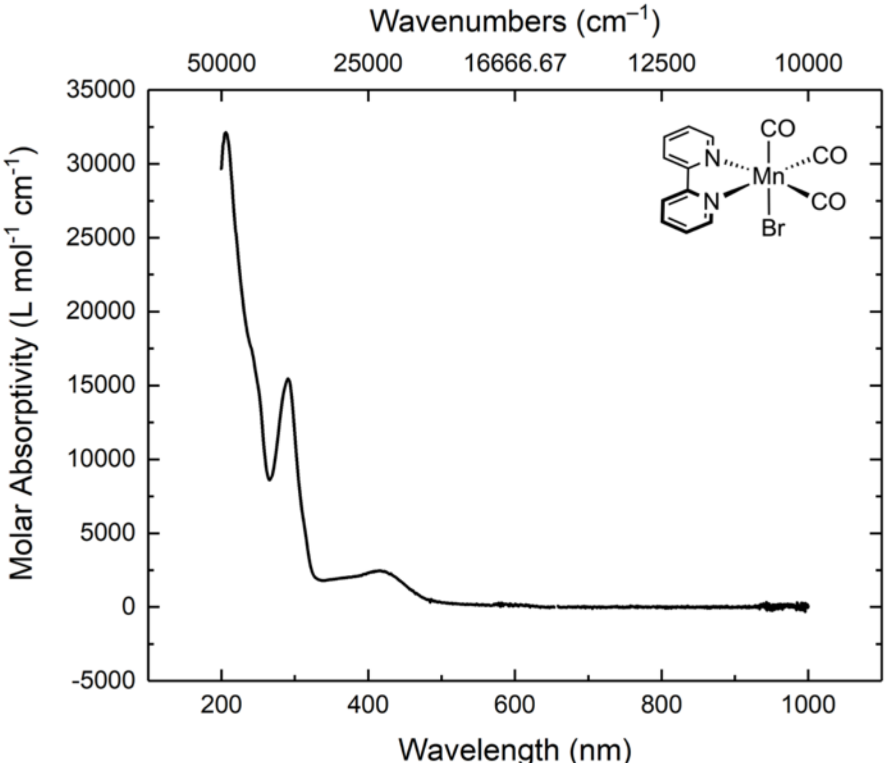

Figure S18: Electronic absorption spectrum of **1** in MeCN**.**

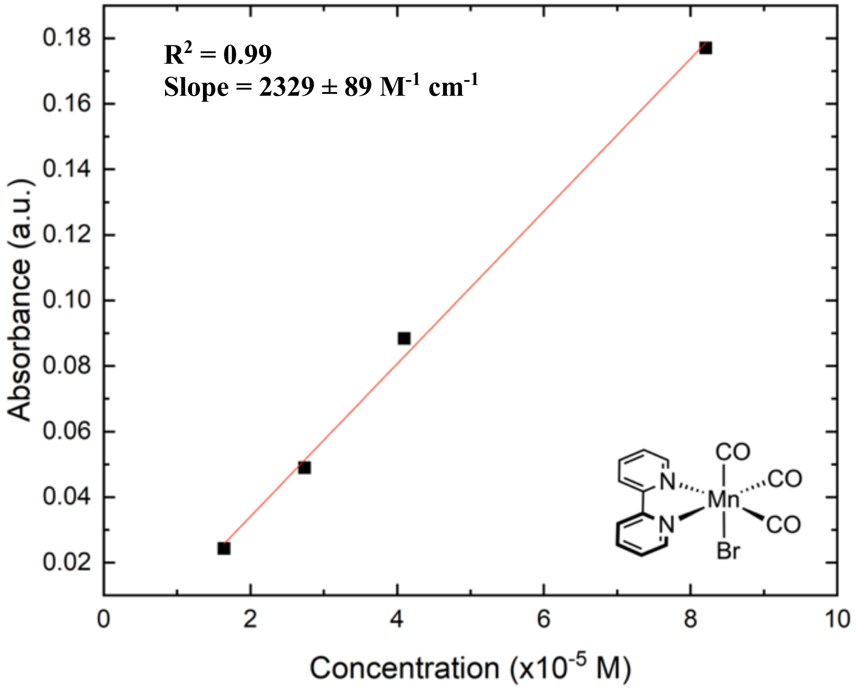

Figure S19: Absorbance vs. concentration plot of complex **1** in MeCN to obtain the molar absorptivity.

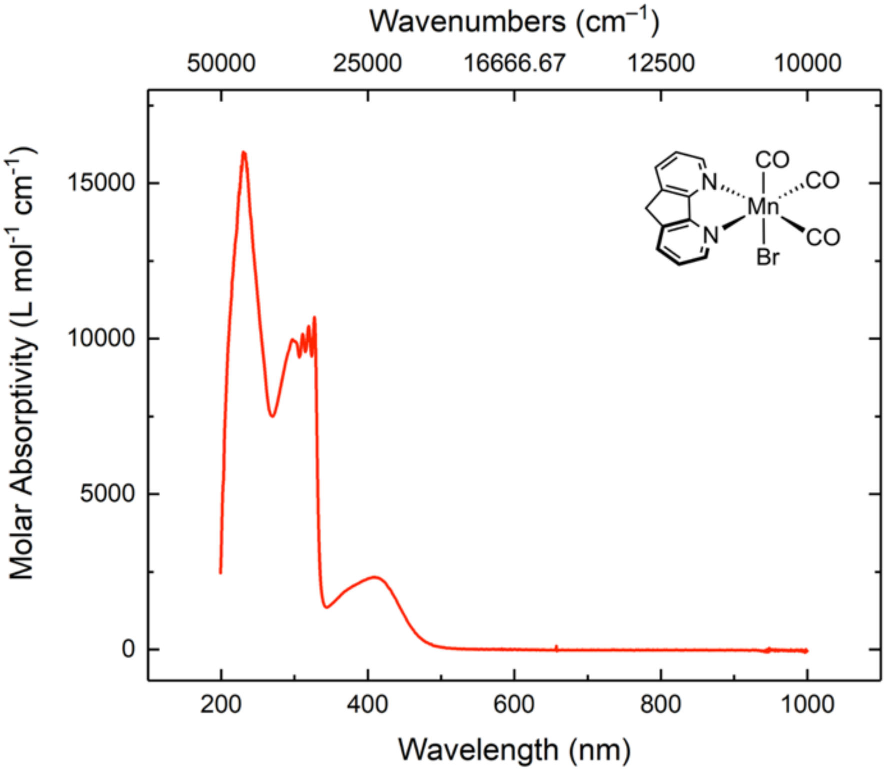

Figure S20: Electronic absorption spectrum of **2** in MeCN**.**

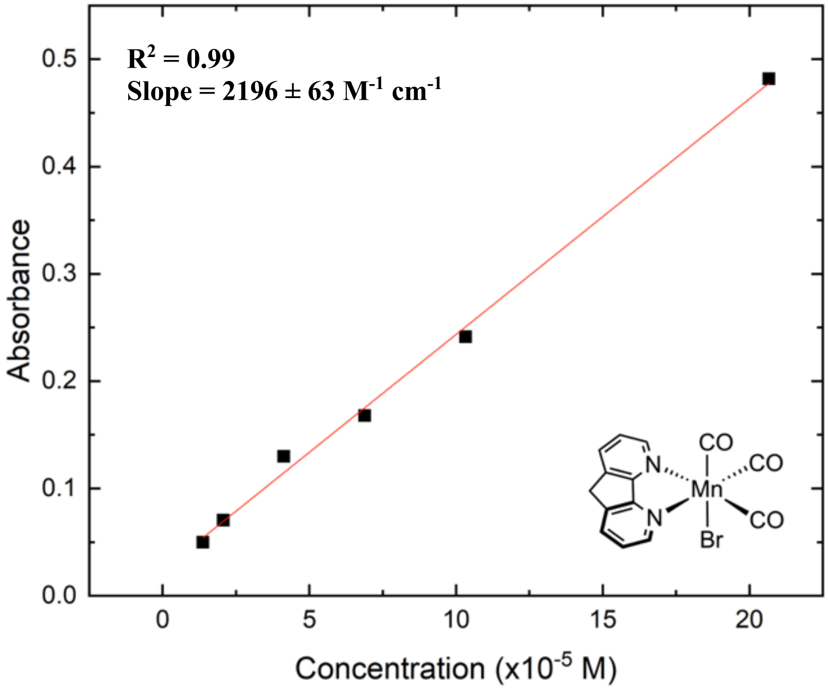

Figure S21: Absorbance vs. concentration plot of complex **2** in MeCN to obtain the molar absorptivity.

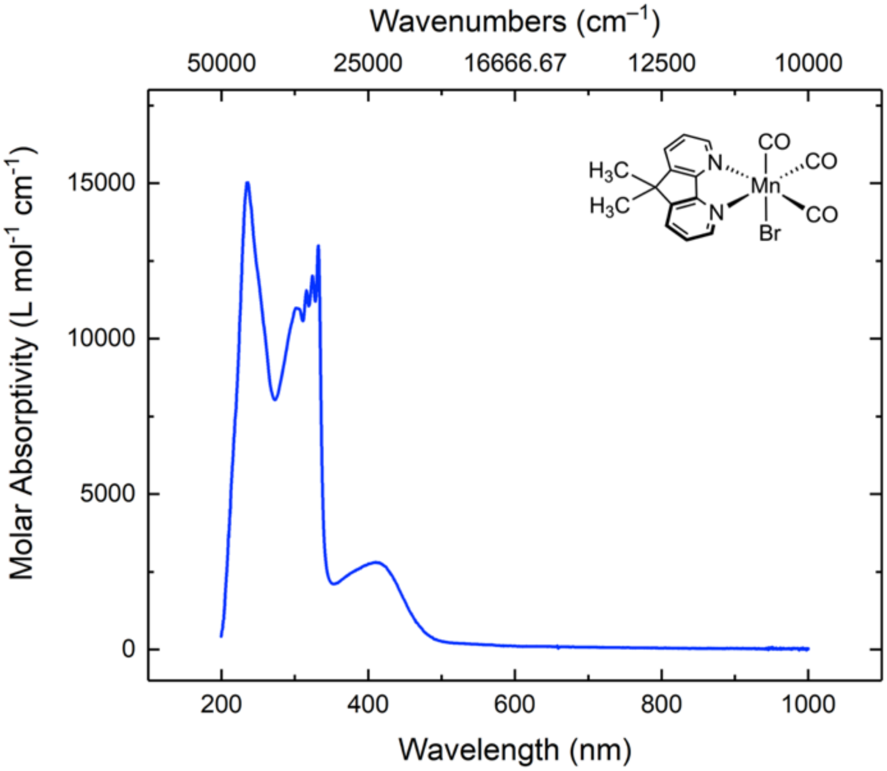

Figure S22: Electronic absorption spectrum of **3** in MeCN**.**

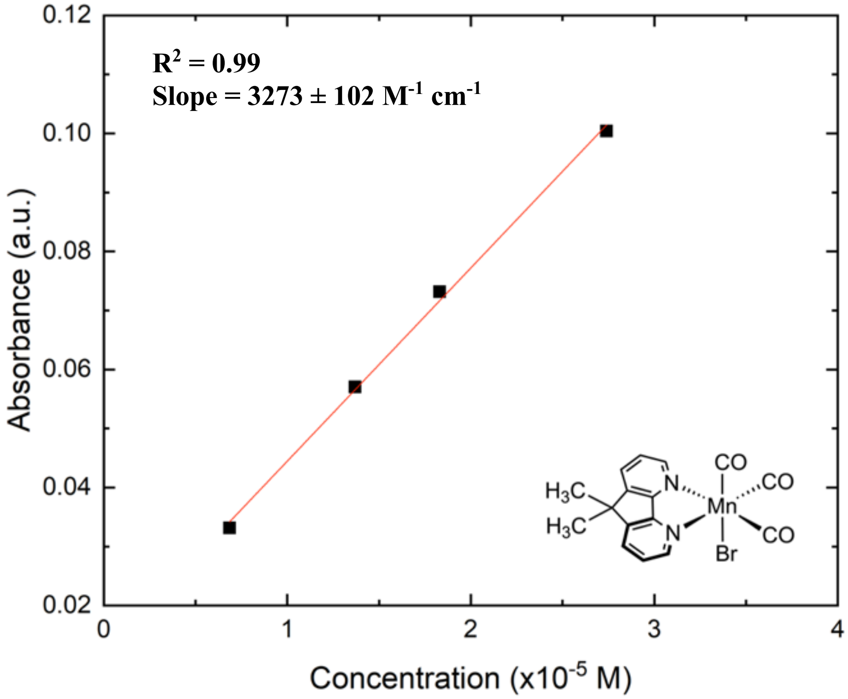

Figure S23: Absorbance vs. concentration plot of complex **3** in MeCN to obtain the molar absorptivity.

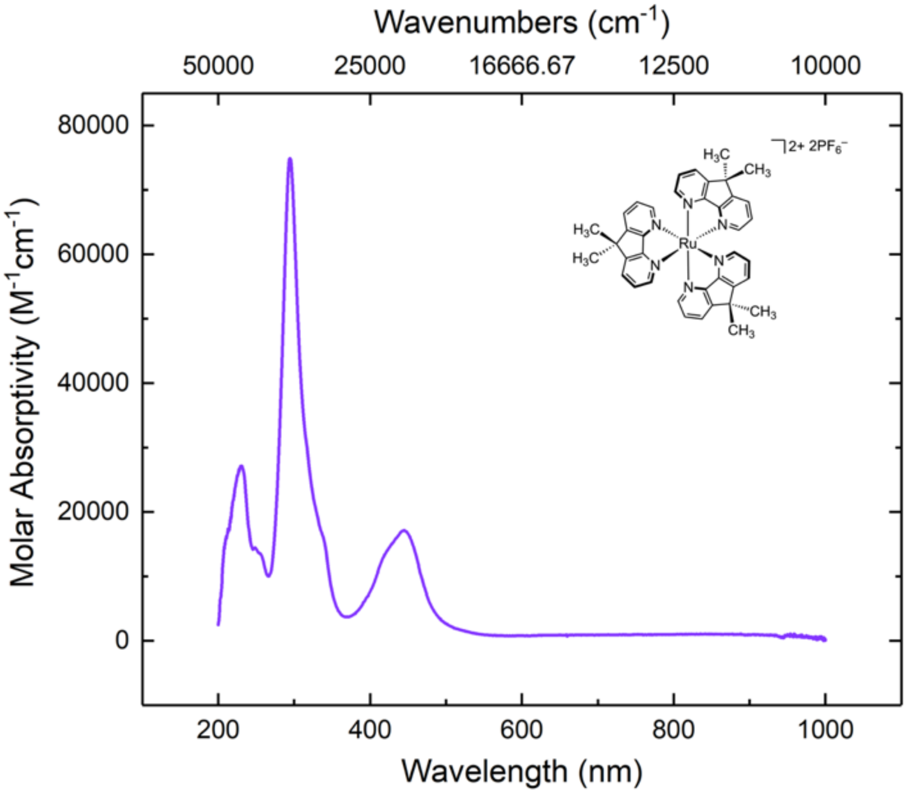

Figure S24: Electronic absorption spectrum of **5** in MeCN.

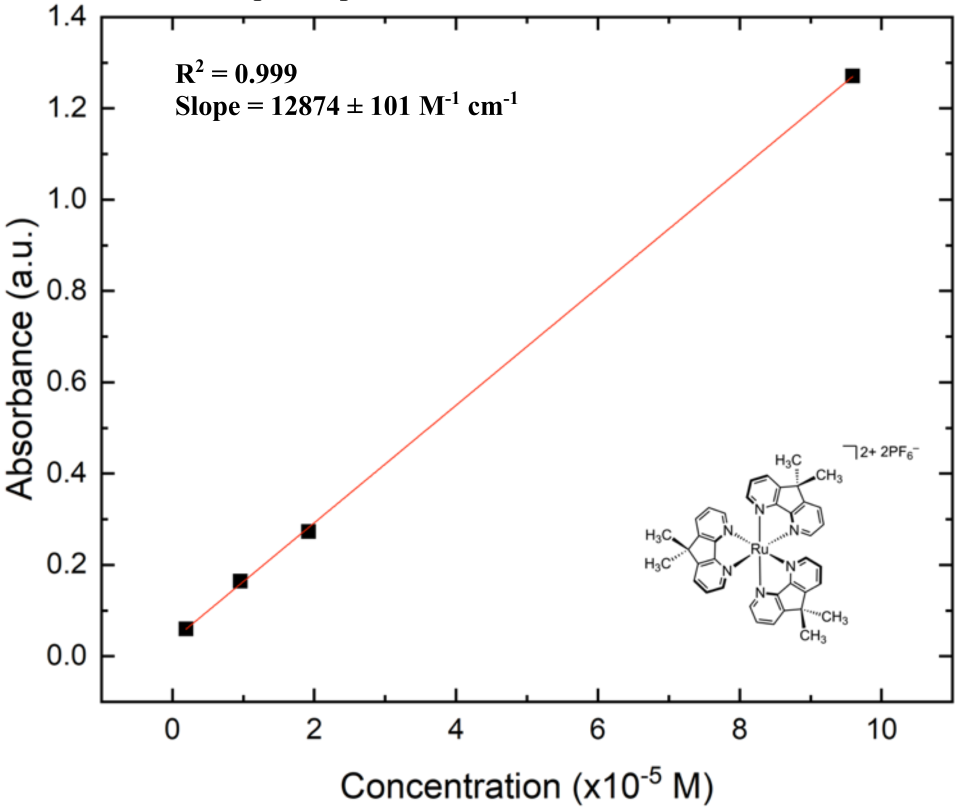

Figure S25: Absorbance vs. concentration plot of complex **5** in MeCN to obtain the molar absorptivity.

Cyclic Voltammetry Data

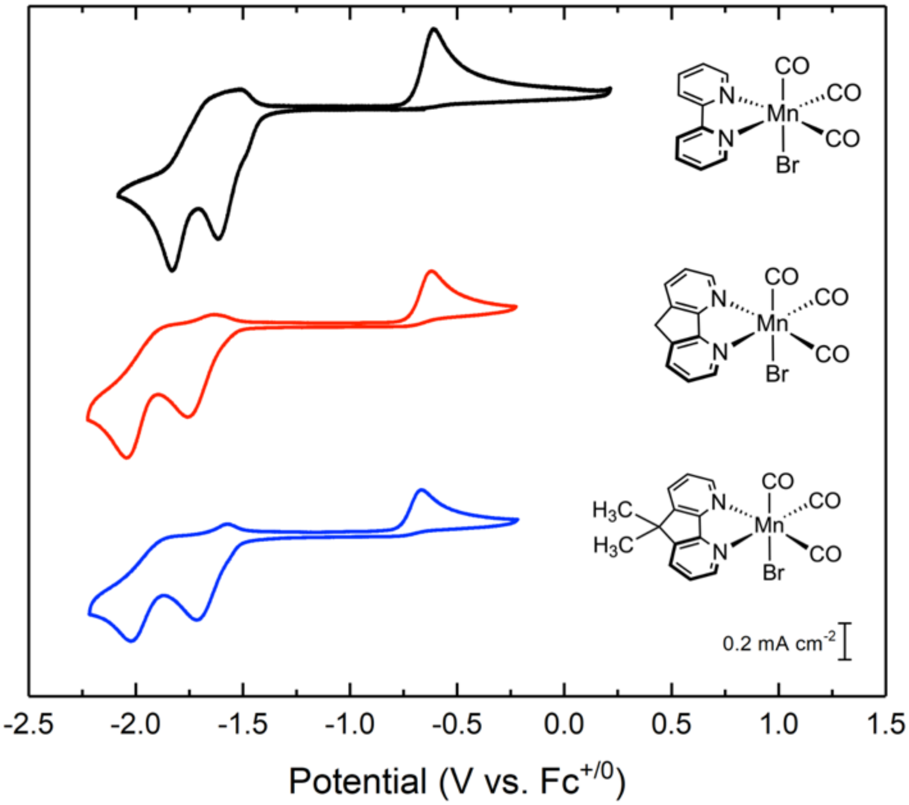

Figure S26: Stacked cyclic voltammograms of **1**, **2**, and **3** in 0.1 M $TBAPF_6$/MeCN solution.

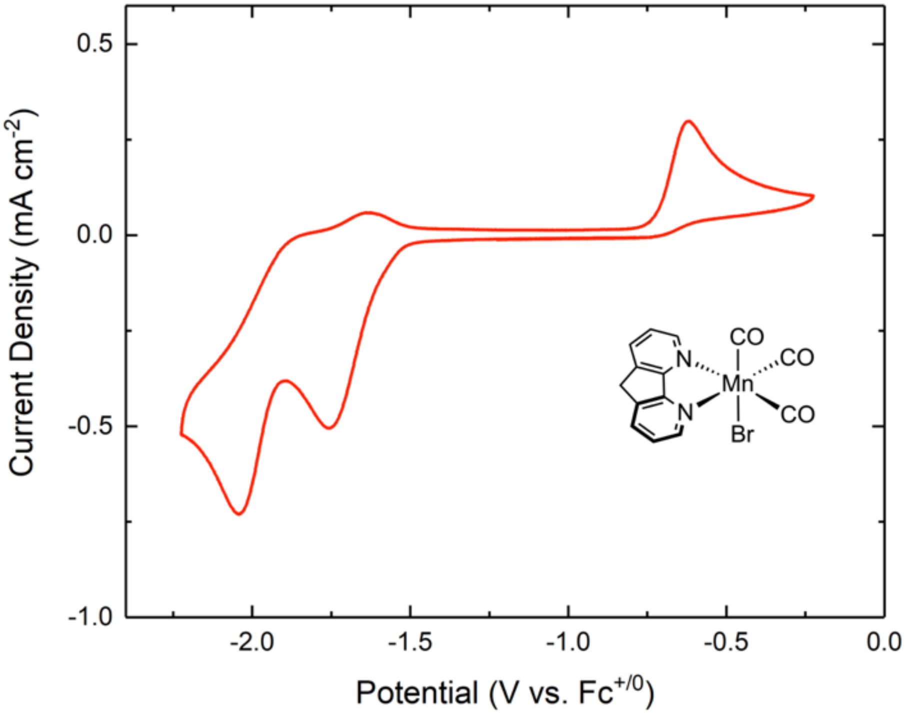

Figure S27: Cyclic voltammogram of **2** in 0.1M $TBAPF_6$/MeCN solution.

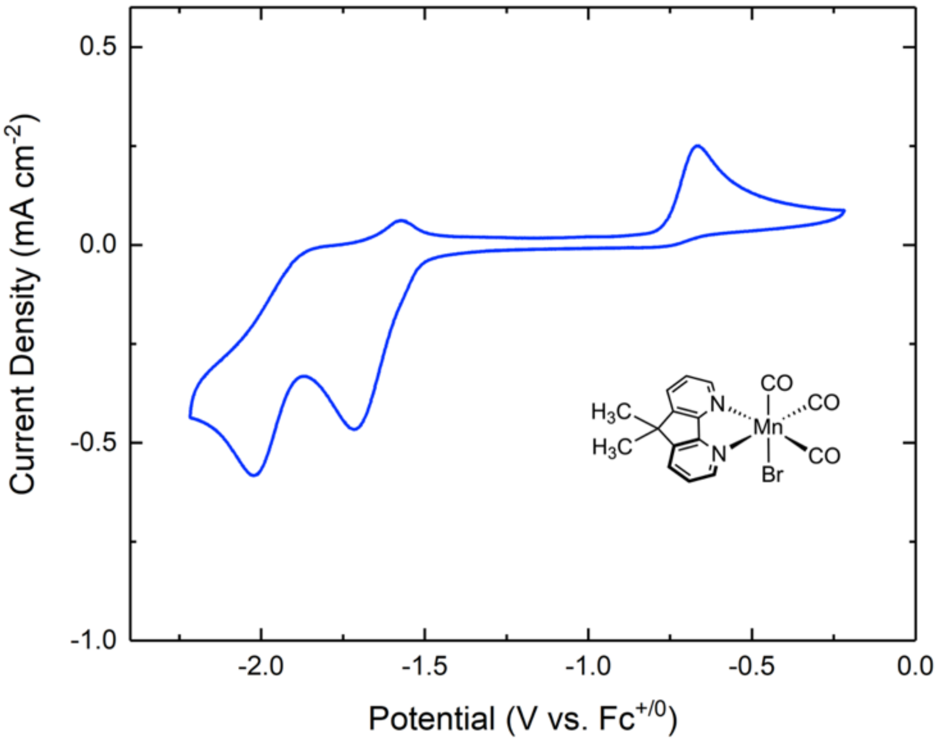

Figure S28: Cyclic voltammogram of **3** in 0.1M $TBAPF_6$/MeCN solution.

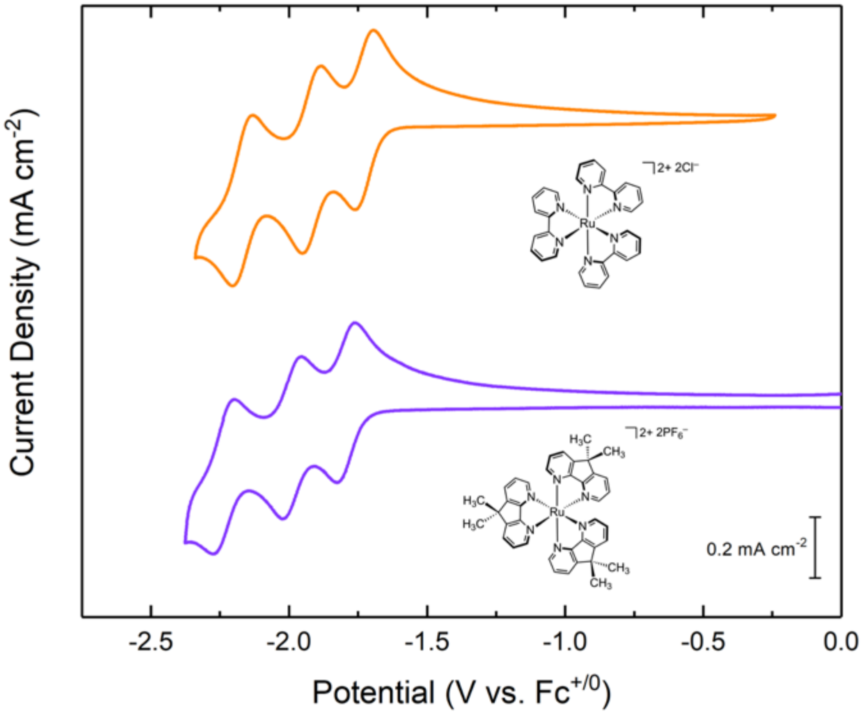

Figure S29: Stacked cyclic voltammograms of **4** and **5** in 0.1M $TBAPF_6$/MeCN solution.

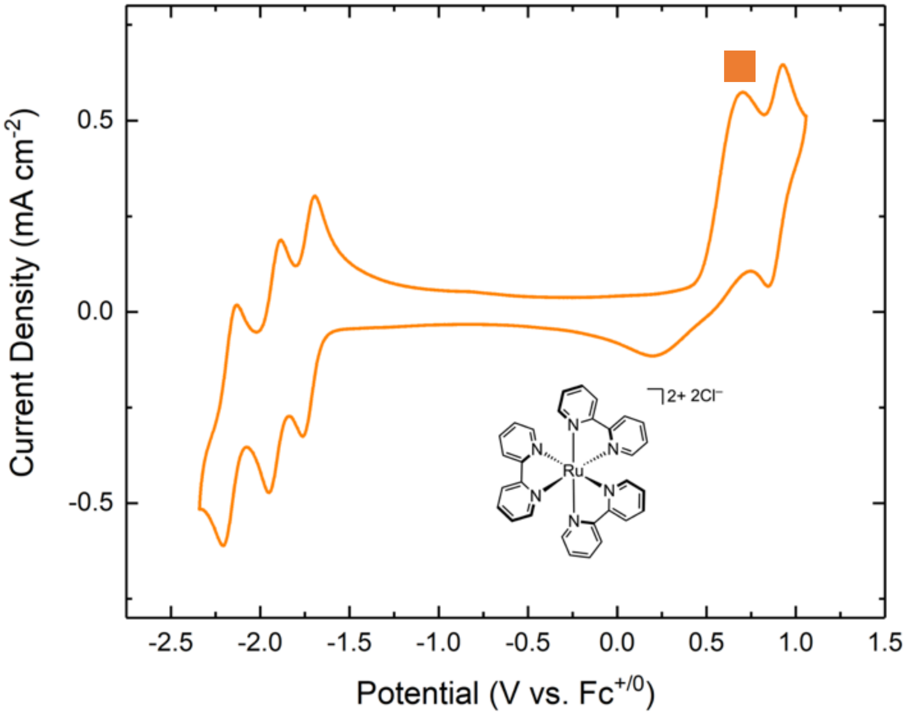

Figure S30: Cyclic voltammograms of **4** in 0.1M $TBAPF_6$/MeCN solution. The orange square (■) indicates chloride oxidation, which is a result of follow up chemical reactivity observed upon reduction.

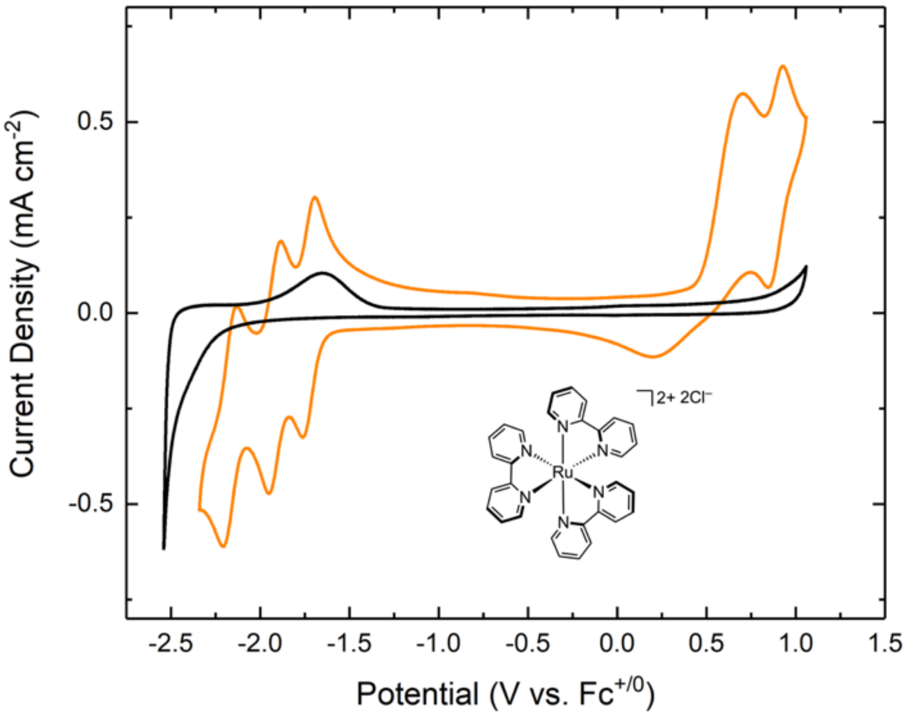

Figure S31: Cyclic voltammograms of **4** overlaid with **blank** in 0.1M $TBAPF_6$/MeCN solution.

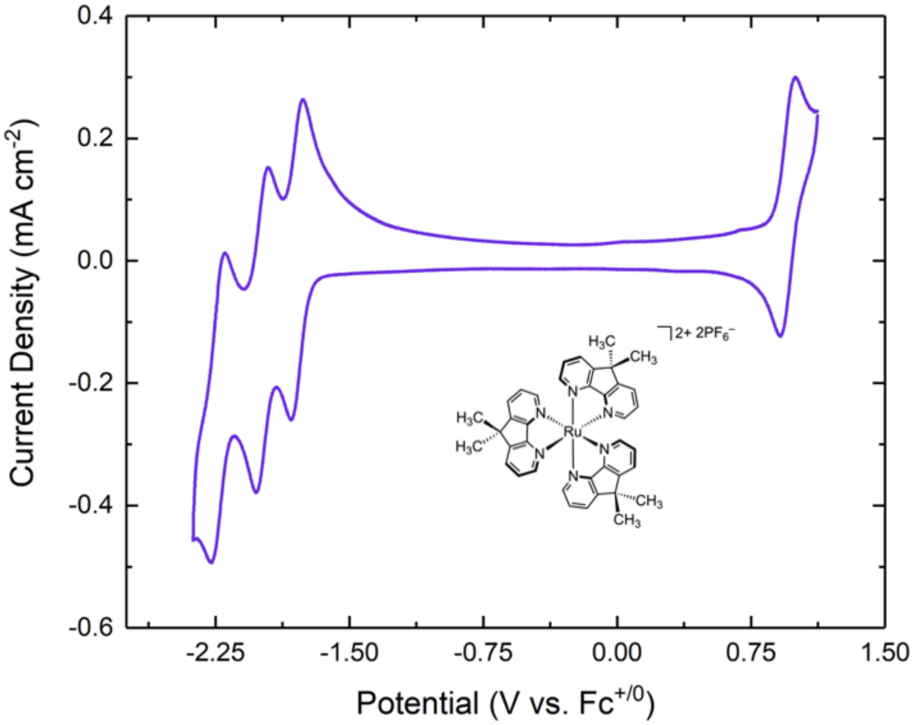

Figure S32: Cyclic voltammograms of **5** in 0.1M $TBAPF_6$/MeCN solution.

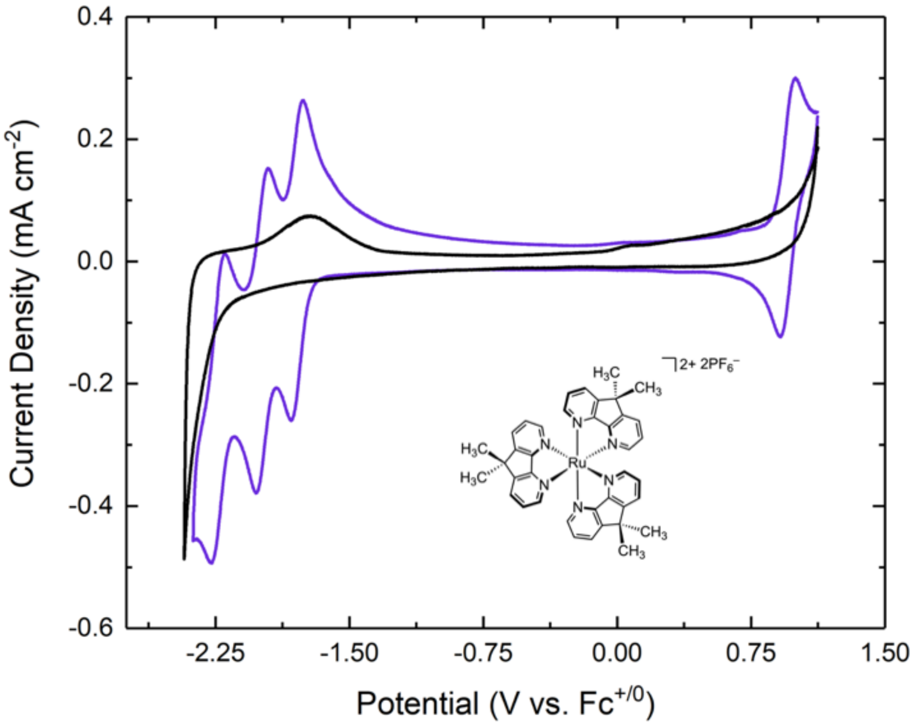

Figure S33: Cyclic voltammogram of **5** overlaid with a **blank** in 0.1M $TBAPF_6$/MeCN solution.

Cyclic Voltammetry with Acid Data

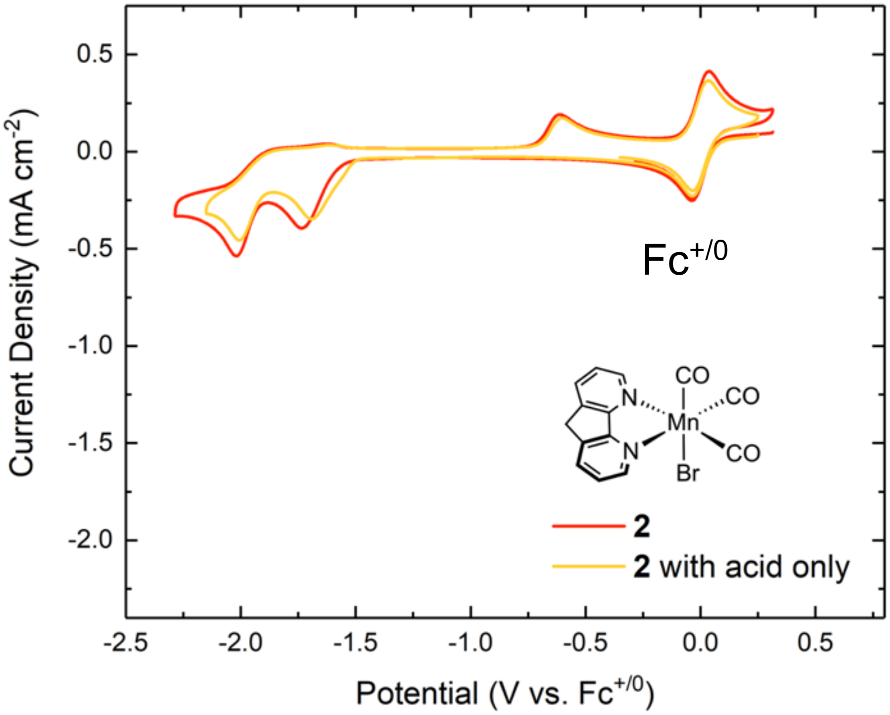

Figure S34: Cyclic voltammetry of **2** (red) in 0.1M $TBAPF_6$/MeCN solution followed by the addition of a 5% H_2O solution (yellow). The diminished current density is a result of dilution.

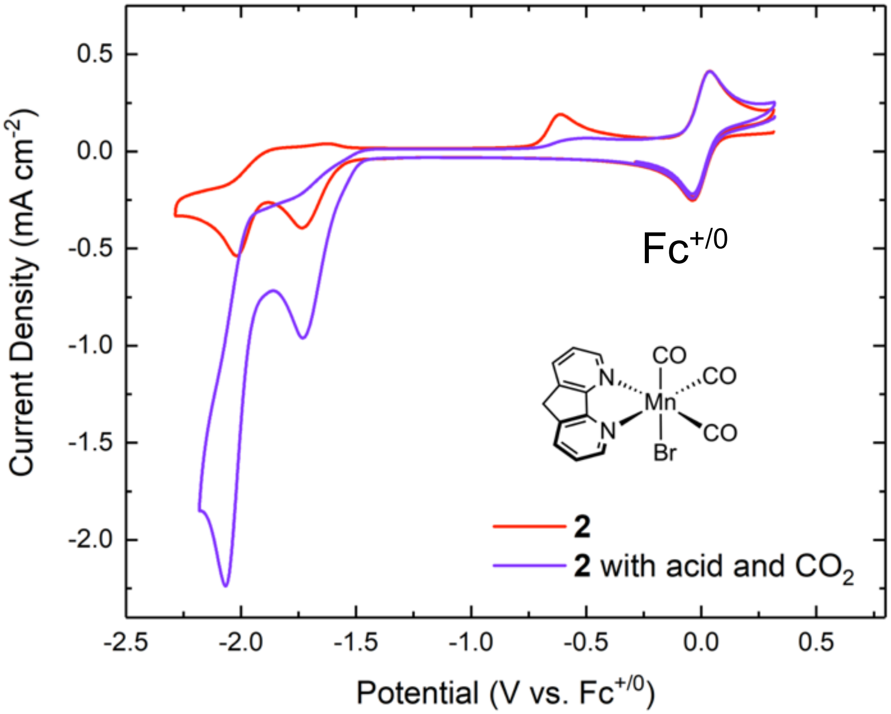

Figure S35: Cyclic voltammetry of **2** (red) in the presence of a 5% H_2O solution and a CO_2 atmosphere (purple).

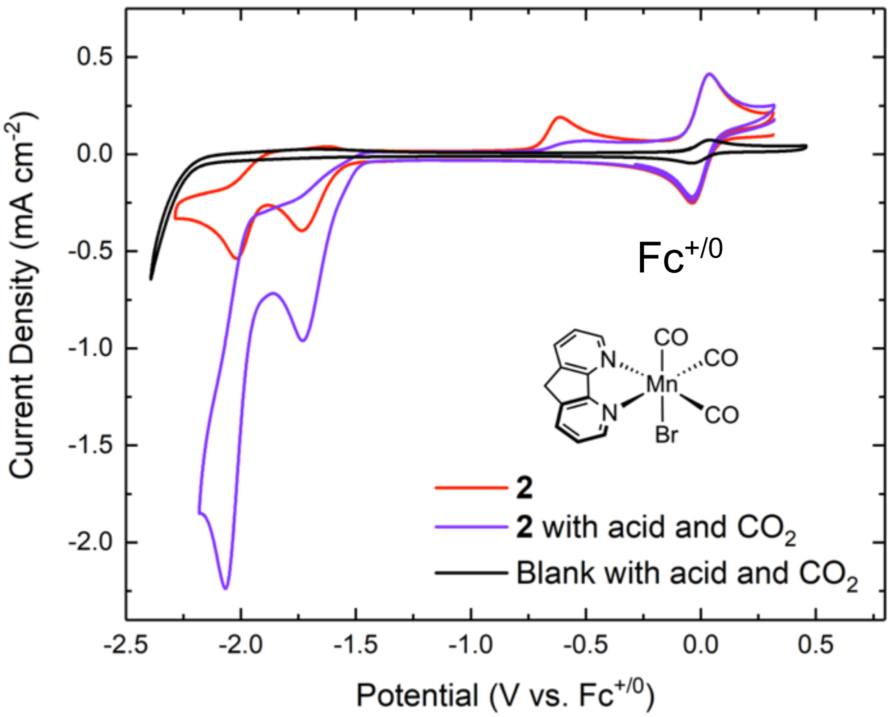

Figure S36: Cyclic voltammetry of **2** (red) and in the presence of a 5% H_2O solution and a CO_2 atmosphere (purple). The black trace illustrates a blank in the presence of a 5% H_2O solution and a CO_2 atmosphere.

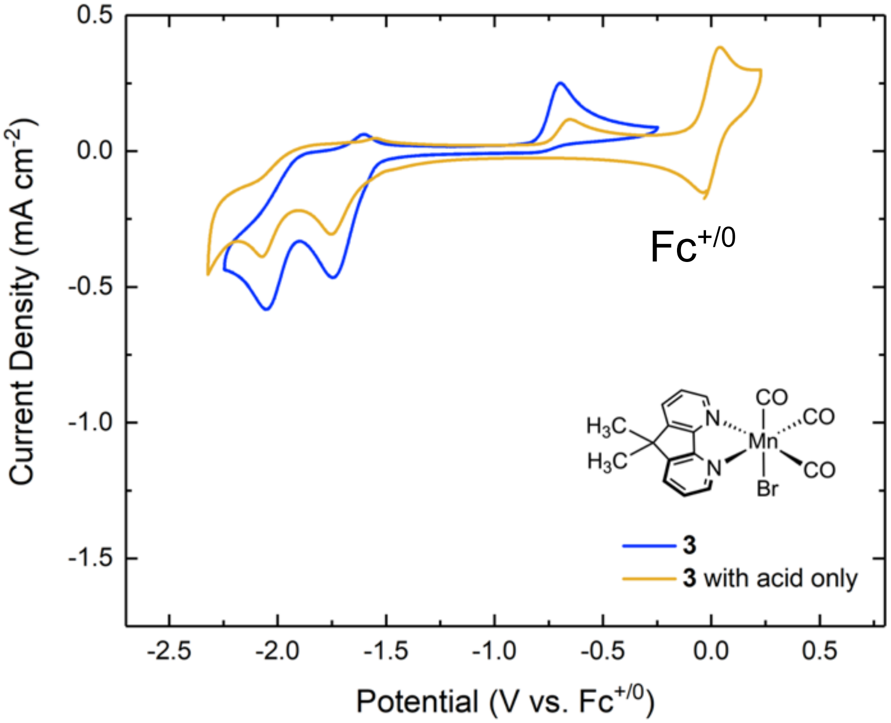

Figure S37: Cyclic voltammetry of **3** (blue) in 0.1M $TBAPF_6$/MeCN solution followed by the addition of a 5% H_2O solution (yellow). The diminished current density is a result of dilution.

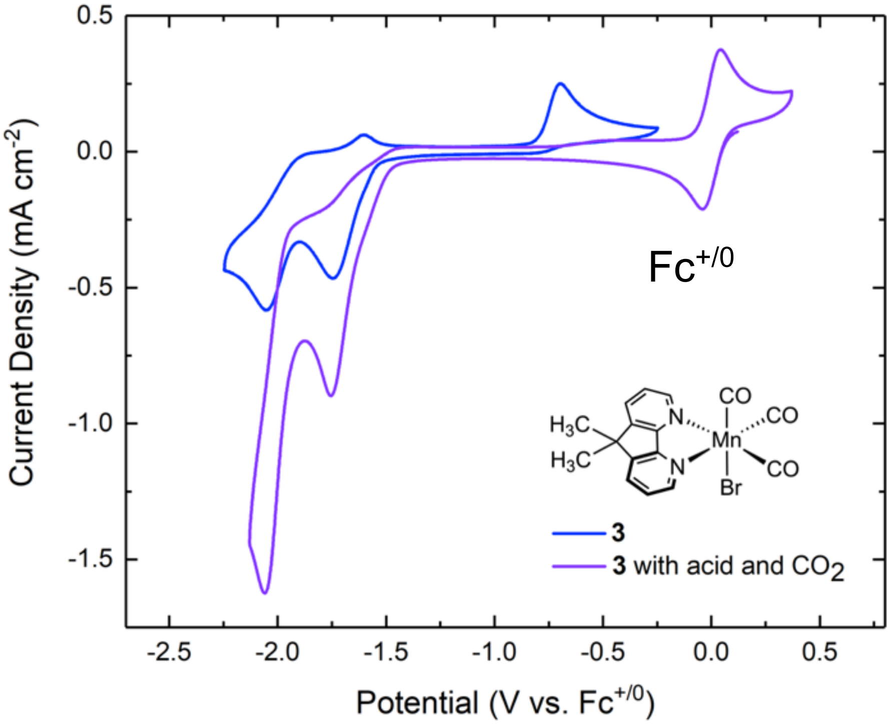

Figure S38: Cyclic voltammetry of **3** (blue) in the presence of a 5% H_2O solution and a CO_2 atmosphere (purple).

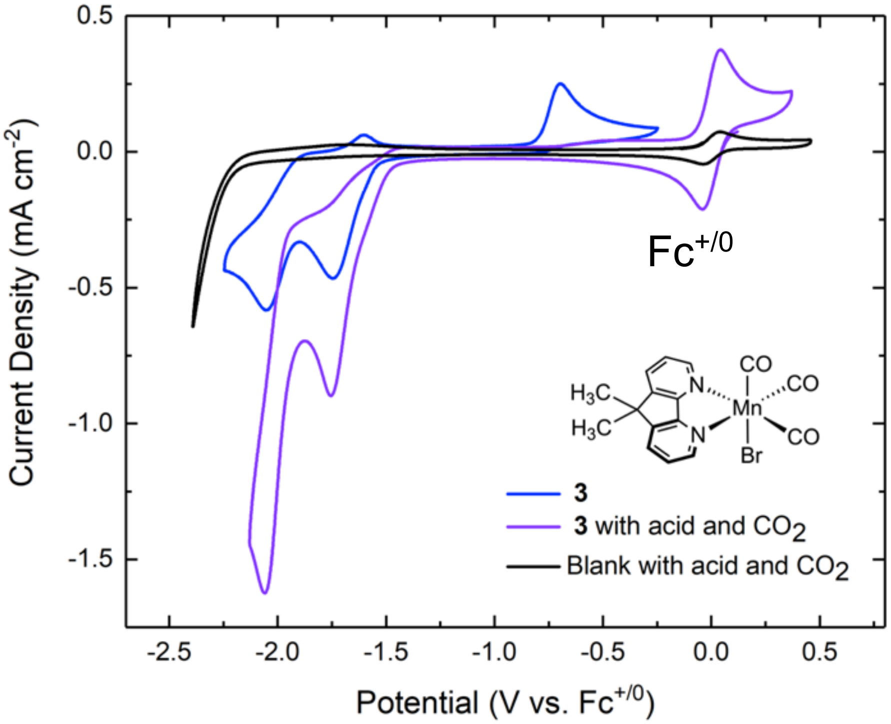

Figure S39: Cyclic voltammetry of **3** (blue) and in the presence of a 5% H_2O solution and a CO_2 atmosphere (purple). The black trace illustrates a blank in the presence of a 5% H_2O solution and a CO_2 atmosphere.

Bulk Electrolysis Data

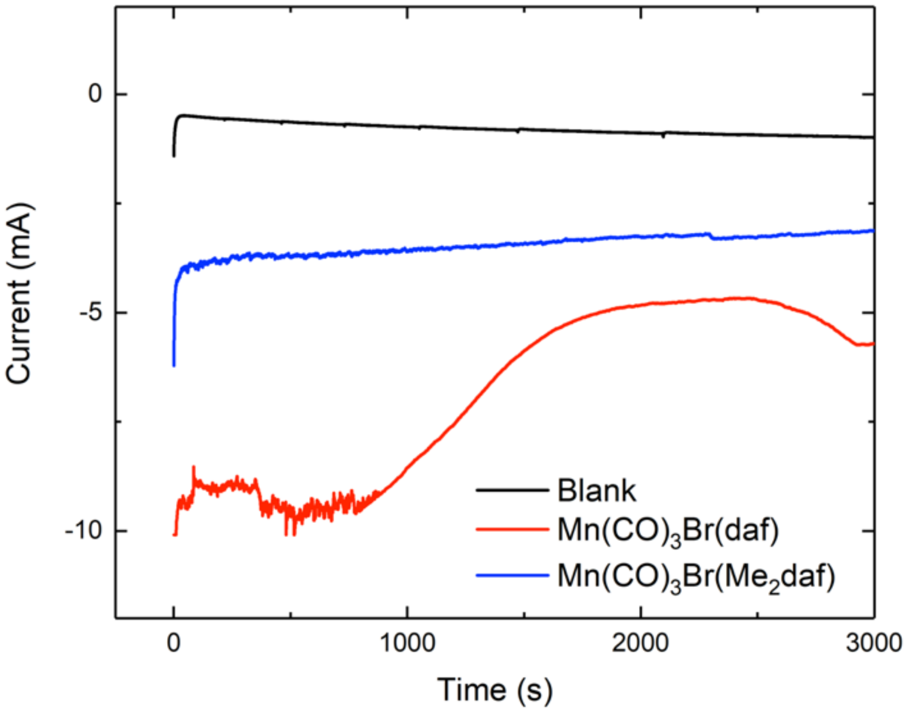

Figure S40: Chronoamperograms demonstrating current passed as a function of time for complexes **2**, **3**, and a **blank** polarized at –2.05 V vs $Fc^{+/0}$ in the presence of a 5% H_2O solution and a CO_2 atmosphere in 0.1M $TBAPF_6$/MeCN electrolyte.

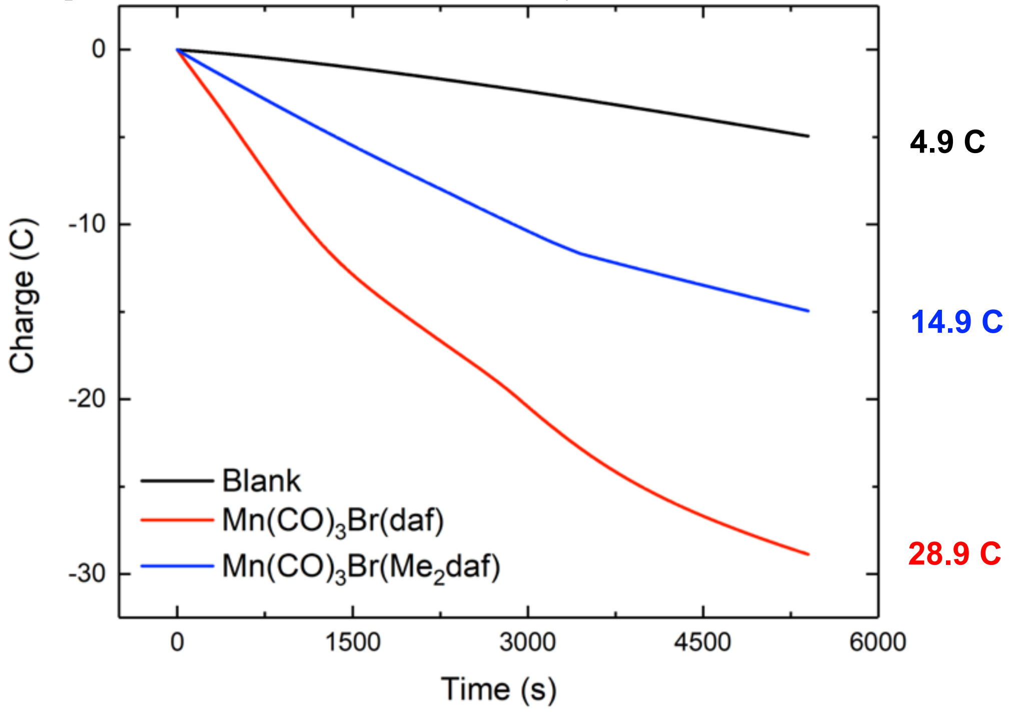

Figure S41: Charge passed as a function of time for complexes **2**, **3**, and a **blank** polarized at –2.05 V vs $Fc^{+/0}$ in the presence of a 5% H_2O solution and a CO_2 atmosphere in 0.1M $TBAPF_6$/MeCN electrolyte.

Table S1: Yield and Faradaic efficiencies for complexes **2** and **3** following a 90 minute controlled potential electrolysis polarized at -2.05 V vs. $Fc^{+/0}$ in in the presence of a 5% H_2O solution and a CO_2 atmosphere in 0.1M $TBAPF_6$/MeCN electrolyte. Water serves as the proton source and Fc is used as the sacrificial reductant.

**Complex**	**H_2 yield (ppm)**	**CO yield (ppm)**	**Faradaic efficiency for H_2 (%)**	**Faradaic efficiency for CO (%)**	**Faradaic Efficiency Total (%)**	**TON H_2**	**TON CO**
**Blank**	1882	271	—	—	—	—	—
**2**	190	8271	1	27	28	0.02	0.82
**3**	137	5484	1	34	35	0.02	0.62

Crystallographic Information

Refinement Details

X-ray Crystallographic Studies for 2 (q51h), 3 (q72j), and 5 (v74e and q36k).

Crystals of **2**, **3**, and **5 (v74e**, **q36k)** were mounted with Paratone N oil in MiTeGen nylon loops under a cold nitrogen stream and placed on a Bruker Proteum diffractometer equipped with two CCD detectors (Apex II and Platinum 135) sharing a common MicroStar microfocus Cu rotating anode generator running at 45 mA and 60 kV (Cu Kα = 1.54178 Å). Complete sets of low temperature (200 K) x-ray diffraction data were obtained for all three compounds using monochromated Cu radiation with the Apex II detector (**2**, **3, 5 (q36k)**) positioned at 50.0 mm and equipped with Helios high brilliance multilayer mirror optics or the Platinum 135 detector (**5 (v74e)**) positioned at 80.0 mm and equipped with Helios high- brilliance multilayer mirror optics. Totals of 7281 (**2**), 1958 (**3**), 2217 (**5(v74e)**), and 2291 (**5 (q36k)**) 1.0°-wide ω- or ϕ-scan frames were collected with counting times of 5-8 seconds (**2**), 10-60 seconds (**3**), 8-30 seconds (**5 (v74e)**), and 4-6 seconds (**5 (q36k)**). Preliminary lattice constants were obtained with SMART in the Bruker Apex2 Software Suite.[1] Integrated reflection intensities for all three compounds were produced using SAINT in the Bruker Apex2 Software Suite.[1] Each data set was corrected empirically for variable absorption effects with SADABS[2] using equivalent reflections. The Bruker software package SHELXTL was used to solve each structure using intrinsic direct methods phasing. Final stages of weighted full-matrix least-squares refinement were conducted using F_o^2 data with SHELXTL[3] or the Olex software package equipped with XL[4]. The relevant crystallographic and structure refinement data for all three structures compounds are given in Table S2.

The final structural model for each structure incorporated anisotropic thermal parameters for all full-occupancy nonhydrogen atoms. The final structural model for each structure incorporated anisotropic thermal parameters for all full-occupancy nonhydrogen atoms. Isotropic thermal parameters were used for all included hydrogen atoms as well as disordered partial-occupancy carbonyl atoms O1′ and C1′ in **3**. Nonmethyl hydrogen atoms were fixed at idealized riding model sp^2- or sp^3-hybridized positions with C-H bond lengths of 0.95 – 0.99 Å. Both methyl groups for the ligands in **3** were incorporated into the structural model as fixed sp^3-hybridized riding-model rigid groups with one methyl hydrogen in the crystallographic mirror plane and C-H bond lengths of 0.96 Å. The six methyl groups in the ligands of **5** (in both structures v74e and q36k) were refined as idealized riding model rigid rotors (with a C-H bond length of 0.98 Å) that were allowed to rotate freely about their C-C bonds in least-squares refinement cycles.

Table S2: Crystal Refinement Data (continues on next page)

	**2 (q51h)**	**3 (q72j)**
**CCDC accession code**	1977431	1994285
**Empirical formula**	$C_{14}H_8BrMnN_2O_3$	$C_{16}H_{12}BrMnN_2O_3$
**Formula weight**	387.07	415.13
**Temperature**	200(2) K	200(2) K
**Wavelength**	1.54178 Å	1.54178 Å
**Crystal system**	Triclinic	Orthorhombic
**Space group**	$P\bar{1}$ - C_i^1 (No. 2)	Cmca - D_{2h}^{18} (No. 64)
**a**	7.1682(7) Å	11.2628(5) Å
**b**	10.0797(10) Å	19.4283(13) Å
**c**	10.5015(7) Å	15.0920(10) Å
**a**	77.757(6)°	90°
**β**	73.507(5)°	90°
**γ**	75.228(6)°	90°
**Volume**	695.47(11) $Å^3$	3302.4(3) $Å^3$
**Z**	2	8
**Density (calculated)**	1.848 g/cm^3	1.670g/cm^3
**Absorption coefficient**	11.193 mm^{-1}	9.473 mm^{-1}
**F(000)**	380	1648
**Crystal size**	0.04 x 0.04 x 0.04 mm^3	0.085 x 0.065 x 0.010 mm^3
**Number of data frames/time**	7281/5-8 seconds	1958/10-60 seconds
**Theta range**	4.44 to 70.32°	4.55 to 70.39°
**Index ranges**	$-8\leq h\leq 8$, $-12\leq k\leq 11$, $-12\leq l\leq 11$	$-12\leq h\leq 13$, $-23\leq k\leq 20$, $-18\leq l\leq 18$
**Reflections collected**	17866	10636
**Independent reflections**	2484 [R_{int} = 0.035]	1635 [R_{int} = 0.073]
**Completeness/θ_{max}**	99.2%/66.00°	99.9%/66.00°
**Absorption correction**	Multi-scan	Multi-scan
**Max. and min. transmission**	1.000 and 0.811	1.000 and 0.659
**Refinement method**	Full-matrix least-squares on F^2	Full-matrix least-squares on F^2
**Data / restraints / parameters**	2484 / 0 /190	1635 / 9 / 128
**Goodness-of-fit on F^2**	1.105	1.181
**Final R indices [I>2σ(I)]**	R_1 = 0.030, wR_2 = 0.081	R_1 = 0.070, wR_2 = 0.179
**R indices (all data)**	R_1 = 0.032, wR_2 = 0.082	R_1 = 0.083, wR_2 = 0.187
**Largest diff. peak and hole**	0.86 and -0.34 $e^-/Å^3$	1.13 and -1.01 $e^-/Å^3$

	5 (v74e)	5 (q36k)
**CCDC accession code**	1982214	2013030
**Empirical formula**	$C_{42.41}H_{36}F_{12}N_6O_4P_2Ru$	$C_{45}H_{45}F_{12}N_9O_{0.5}P_2Ru$
**Formula weight**	1084.70	1110.91
**Temperature**	200(2) K	200(2) K
**Wavelength**	1.54178 Å	1.54178 Å
**Crystal system**	Monoclinic	Monoclinic
**Space group**	$P2_1$ - C_2^2 (No. 4)	$P2_1$ - C_2^2 (No. 4)
**a**	11.7917(10) Å	11.7769(5) Å
**b**	19.7081(14) Å	19.6256(8) Å
**c**	12.3211(11) Å	12.3972(5) Å
**a**	90°	90°
**β**	110.665(5)°	110.8508(17)°
**γ**	90°	90°
**Volume**	2697.1(4) $Å^3$	2677.70(19) $Å^3$
**Z**	2	2
**Density (calculated)**	1.345 g/cm^3	1.378 g/cm^3
**Absorption coefficient**	3.682 mm^{-1}	3.661 mm^{-1}
**F(000)**	1093	1128
**Crystal size**	0.540 x 0.097 x 0.060 mm^3	0.204 x 0.051 x 0.027 mm^3
**Number of data frames/time**	2217/8-30 seconds	2291/4-6 seconds
**Theta range**	4.44 to 68.72°	3.82 to 70.23°
**Index ranges**	$-13 \leq h \leq 13$, $-16 \leq k \leq 23$, $-14 \leq l \leq 14$	$-14 \leq h \leq 13$, $-21 \leq k \leq 23$, $-14 \leq l \leq 14$
**Reflections collected**	17556	19973
**Independent reflections**	7199 [R_{int} = 0.069]	7988 [R_{int} = 0.053]
**Completeness/θ_{max}**	99.0%/66.00°	99.5%/66.00°
**Absorption correction**	Multi-scan	Numerical face-indexed
**Max. and min. transmission**	1.000 and 0.500	1.000 and 0.322
**Refinement method**	Full-matrix least-squares on F^2	Full-matrix least-squares on F^2
**Data / restraints / parameters**	7199 / 43 / 640	7988 / 1 / 641
**Goodness-of-fit on F^2**	1.057	1.072
**Final R indices [I>2σ(I)]**	R_1 = 0.112, wR_2 = 0.294	R_1 = 0.053, wR_2 = 0.141
**R indices (all data)**	R_1 = 0.125, wR_2 = 0.309	R_1 = 0.055, wR_2 = 0.143
**Largest diff. peak and hole**	1.84 and -1.05 $e^-/Å^3$	1.18 and -0.95 $e^-/Å^3$

Special Refinement Details for 2.

No special refinement was required

Full Solid-state Structure of 2.

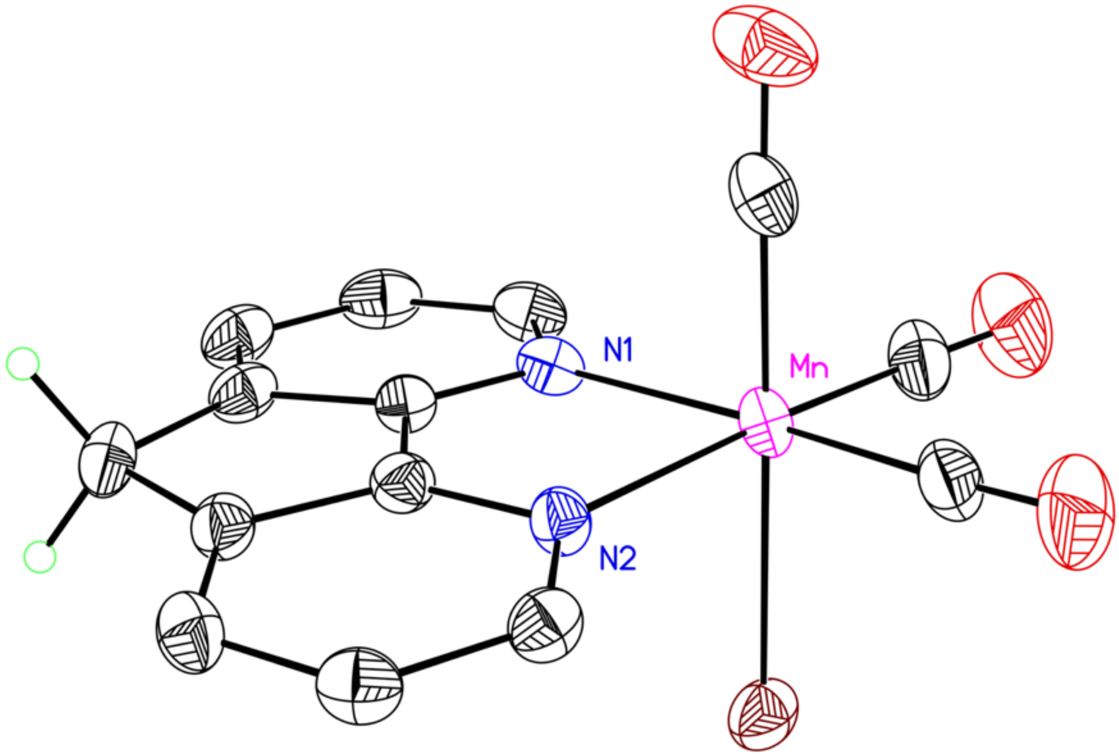

Figure S42: Full solid-state structure of **2**. Hydrogen atoms are omitted for clarity except on the methylene backbone. Displacement ellipsoids shown at the 50% probability level.

Special Refinement Details for 3.

Since Br and trans-coordinated CO ligands bonded to Mn have similar sizes and shapes, it is not surprising that the C_s- $Mn(CO)_3(N_2C_{13}H_{12})Br$ molecule (**3**) might pack in a disordered fashion with the Br and trans-coordinated CO ligands interchanged 31% of the time. This disorder necessitated mild bond length and angle restraints for this disordered group of atoms. The Mn-Br, Mn-Br′, Mn-O1, Mn-O1′, Mn-C1, Mn-C1′, C1-O1, C1′-O1′and C2-O2 bond lengths in **3** were all mildly restrained to have values which were appropriate multiples of the Mn-Br bond length that was included as a free variable in the least-squares refinement and refined to a final value of 2.406(7)Å. When the isotropic thermal parameter for C1′ refined to an unrealistically high value, it was fixed at a value equal to the average of Mn and O1′.

Solid-state Structure of 3.

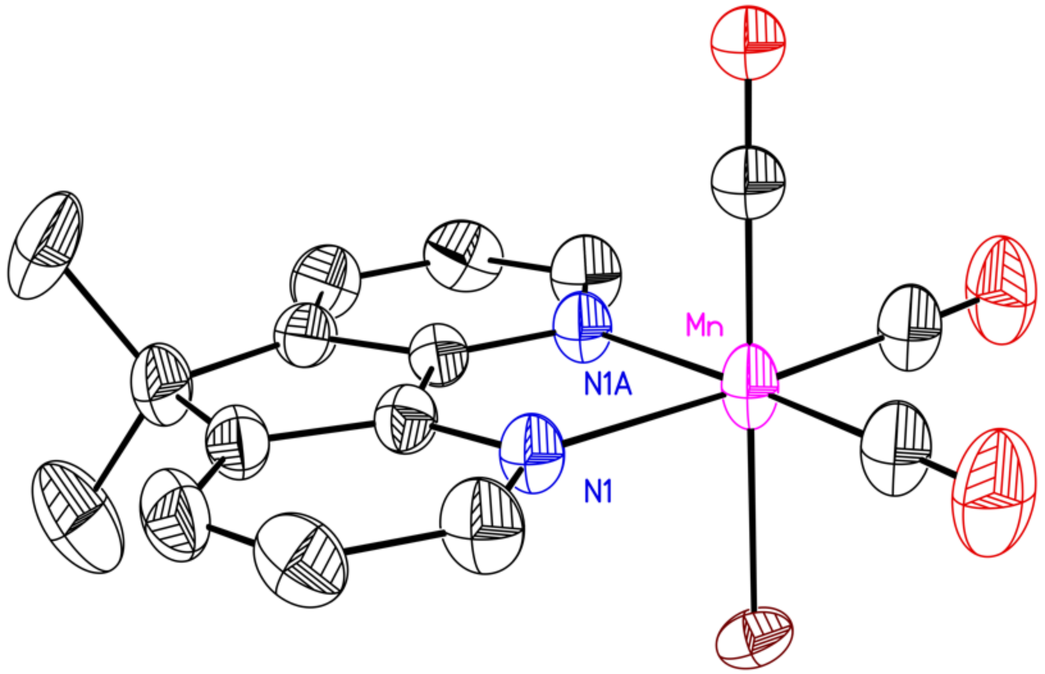

Figure S43: Solid-state structure of **3**. Hydrogen atoms, a second image of the molecule of **3** present in the asymmetric unit is omitted for clarity. Displacement ellipsoids shown at the 50% probability level.

Full Solid-state Structure of 3.

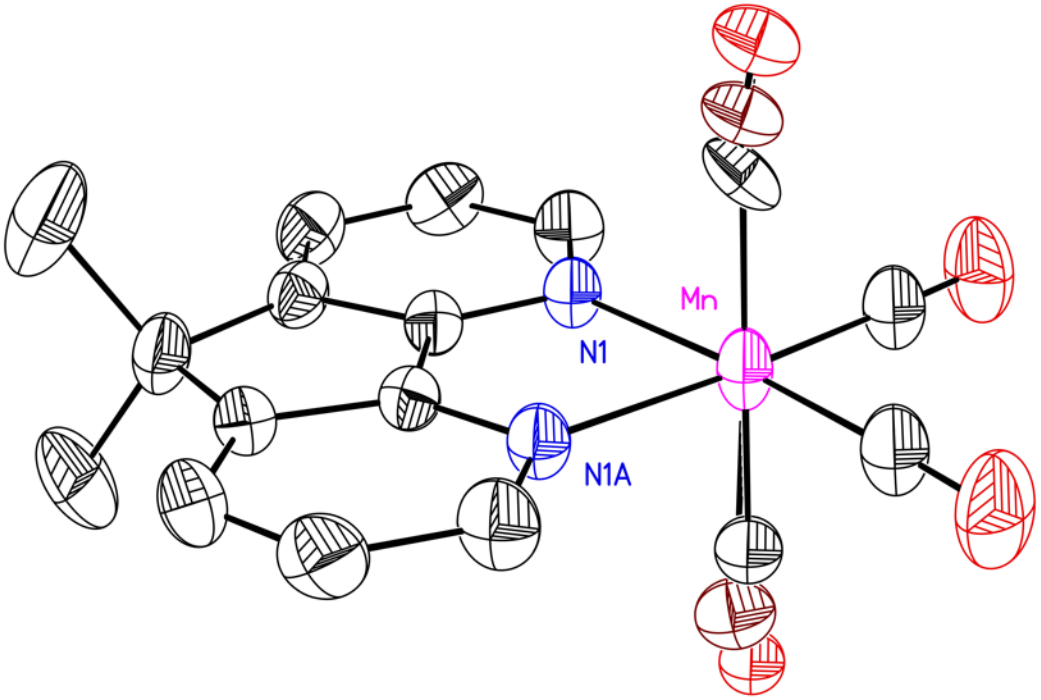

Figure S44: Full solid-state structure of **3**. Hydrogen atoms are omitted for clarity. Displacement ellipsoids shown at the 50% probability level.

Special Refinement Details for 5 (v74e).

The solvent molecules of crystallization in the first structure of **5 (v74e)** are disordered and hydrogen atoms were not included for them in the structural model. A methanol molecule (atoms O3S and C4S) is present 41% of the time and a nearby water molecule (oxygen O1W) is present the remaining 59% of the time. A second water molecule is 77/23 disordered over two closely-spaced sites (oxygen atoms O2W and O2W'). We note explicitly here that no rigorous H-bonding scheme could be detected for these solvent molecules of crystallization in this crystal. Reliable structural assignments for atoms of the solvent molecules of crystallization could therefore not be made. However, it was felt that these details would not have a major impact on the structural assignment for the $[Ru(Me_2daf)_3]^{2+}$ core and disordered solvent atoms were assigned based on solvents known to possibly be present. Their occupancy factors were reduced to give more reasonable thermal parameters. A subsequent data set (**q36k**, *vide infra*) for freshly prepared and crystallized **5** revealed that the disordered solvent for **v74e** was probably acetonitrile and water.

Additionally, the structure of **5 (v74e)** was refined as a 52/48 racemic twin.

Solid-state Structure of 5 (v74e).

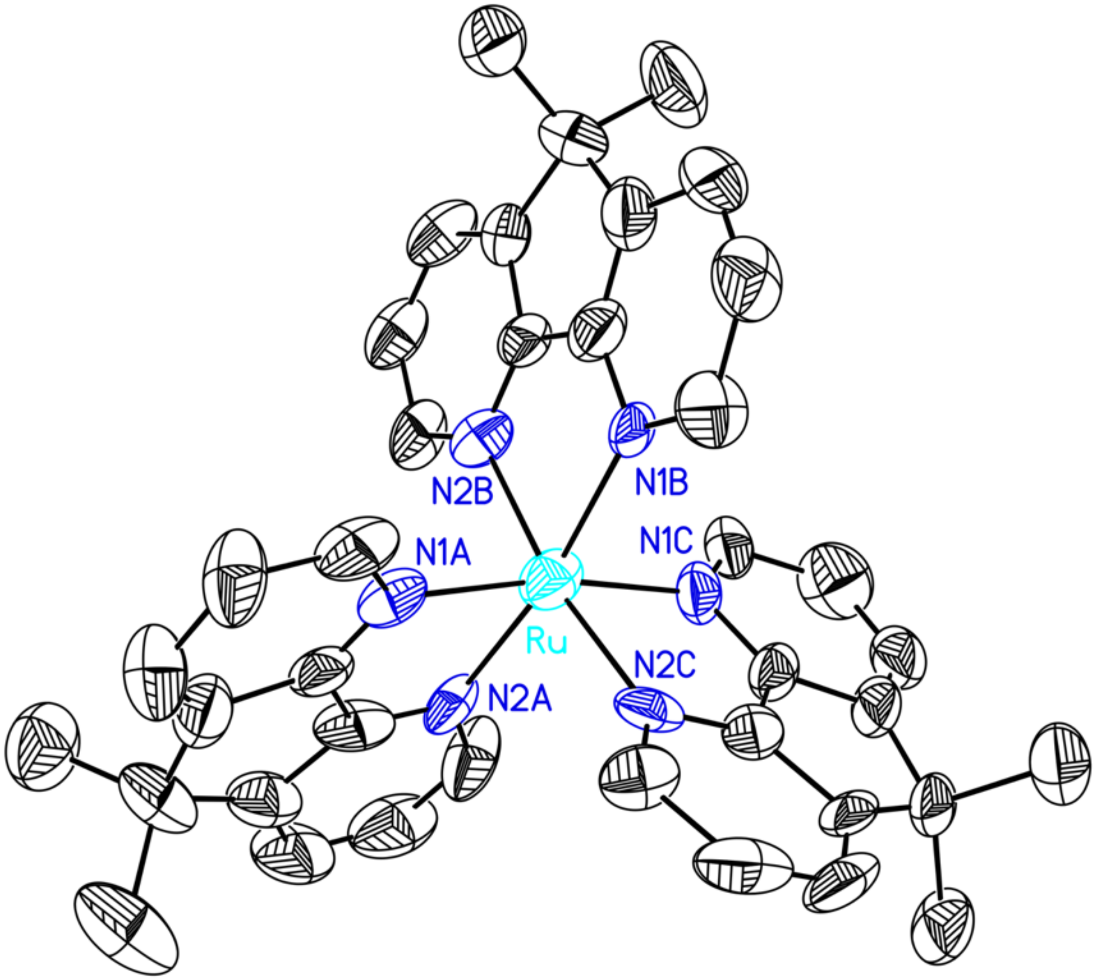

Figure S45: Solid-state structure of **5 (v74e)**. Hydrogen atoms and co-crystalized water, methanol, and ethanol are omitted for clarity. Displacement ellipsoids shown at the 50% probability level.

Full Solid-state Structure of 5 (v74e).

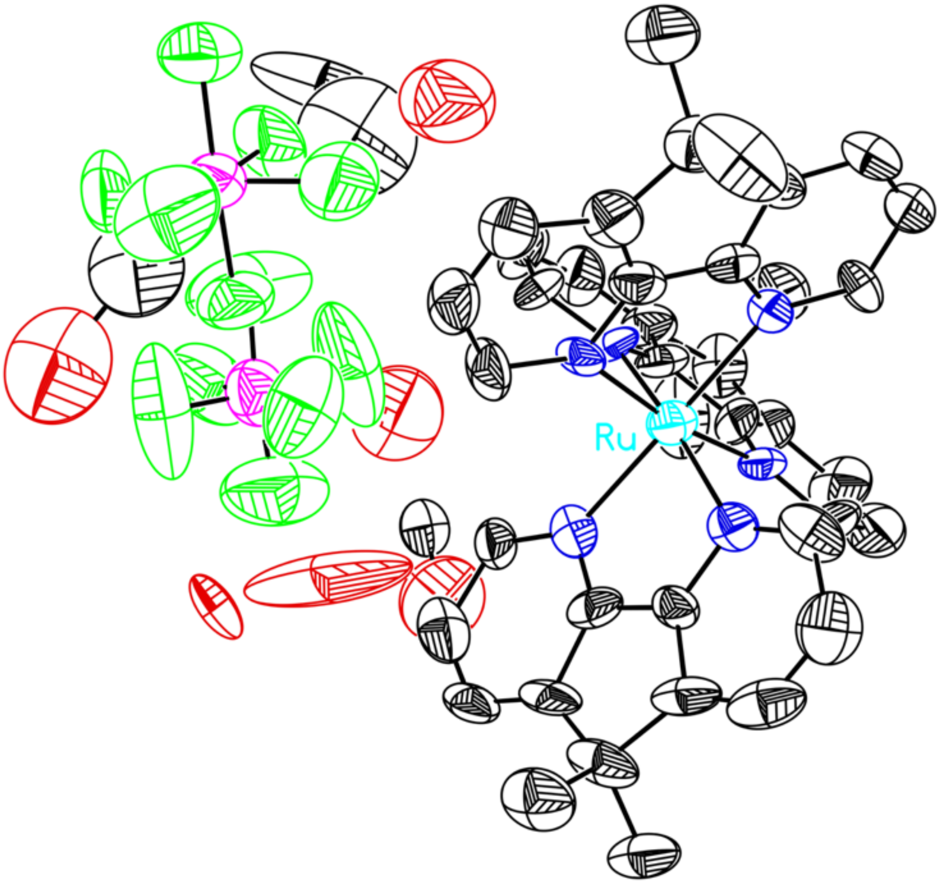

Figure S46: Full solid-state structure of **5 (v74e)**. Hydrogen atoms omitted for clarity. Displacement ellipsoids shown at the 50% probability level. Atoms attributable to co-crystalized water, methanol, and ethanol are shown.

Special Refinement Details for 5 (q36k).

The water molecule of crystallization appears to be present only part of the time. So, the occupancy factor of oxygen atom O1W was fixed at 0.50 and its covalently-bonded hydrogen atoms were not included in the structural model. The structure was refined as a 94/6 racemic twin.

Additionally, this structure of **5 (q36k)** was refined as a 94/6 racemic twin.

Solid-state Structure of 5 (q36k).

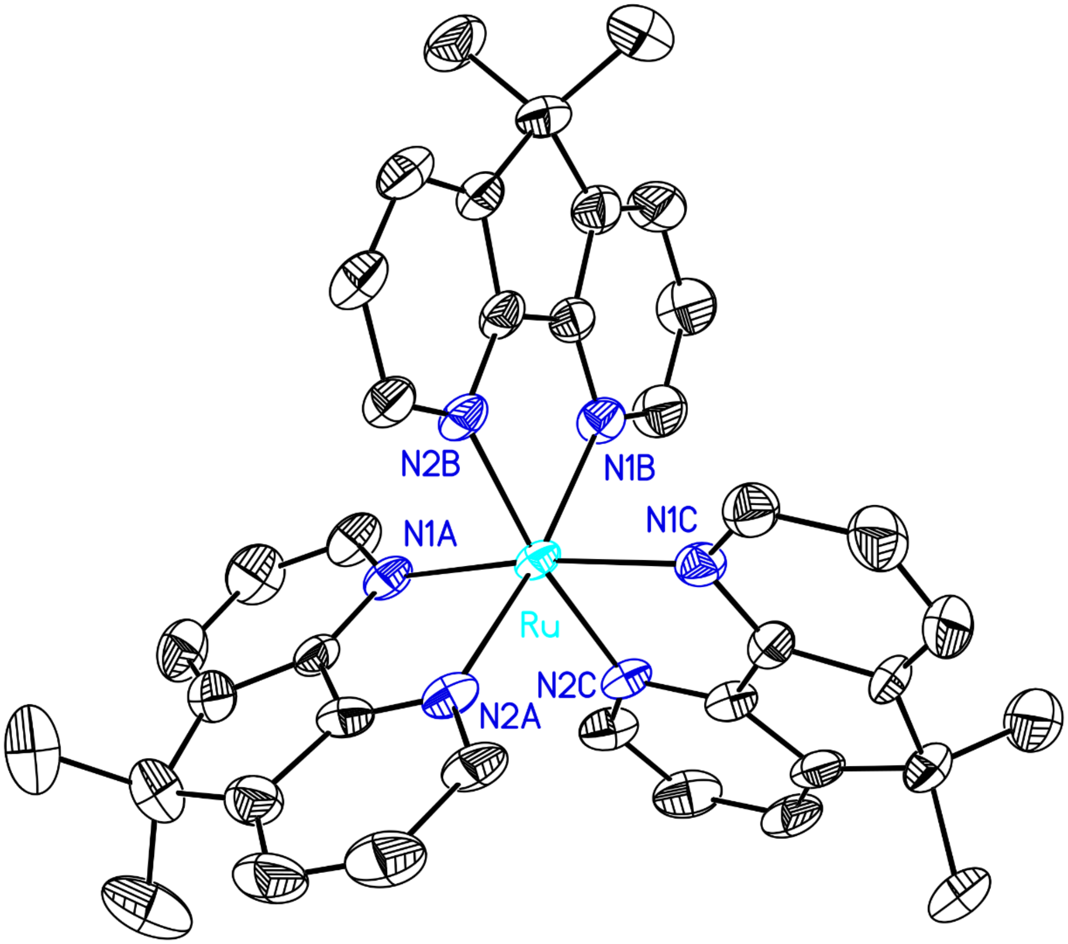

Figure S47: Solid-state structure of **5 (q36k)**. Displacement ellipsoids shown at the 50% probability level. Hydrogen atoms, atoms attributable to three co-crystalized acetonitrile molecules, and a disordered solvent water molecule are omitted for clarity.

Full Solid-state Structure of 5 (q36k).

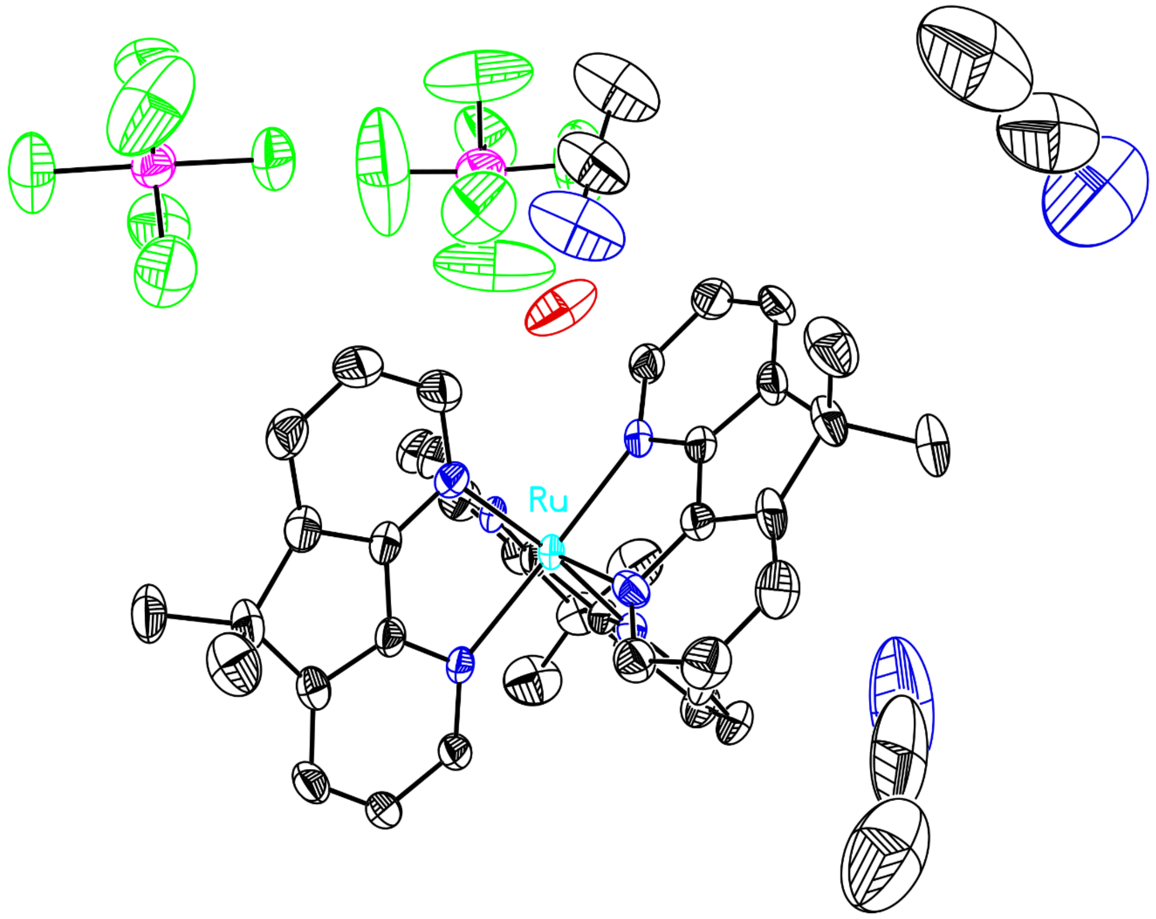

Figure S48: Full solid-state structure of **5 (q36k)**. Hydrogen atoms omitted for clarity. Displacement ellipsoids shown at the 50% probability level. Atoms attributable to three co-crystalized acetonitrile molecules and a disordered solvent water molecule are also present.

Table S3: Selected bond lengths in Ångstroms (Å).

	**5 (v74e)**	**5 (q36k)**
**Ru–N**	2.090(14)	2.104(5)
	2.143(15)	2.108(5)
	2.150(14)	2.114(6)
	2.154(14)	2.123(6)
	2.158(12)	2.125(5)
	2.159(15)	2.130(5)
**Ru–N (avg.)**	**2.142(34)**	**2.117(13)**
**C5-C6**	1.39(2)	1.416(9)
	1.40(3)	1.420(9)
	1.43(2)	1.425(9)
**C5-C6 (avg.)**	**1.41(4)**	**1.420(16)**

Table S4: Selected bond angles in degrees (º).

	**5 (v74e)**	**5 (q36k)**
**N–Ru–N**	79.5(5)	82.2(2)
	81.7(5)	83.1(2)
	82.1(6)	83.3(2)
**N–Ru–N (avg.)**	**81.1(9)**	**82.9(3)**
**CH_3–C–CH_3**	108(2)	109.4(7)
	112(2)	109.9(7)
	109.5(17)	111.9(8)
**CH_3–C–CH_3** (avg.)	**110(3)**	**110.4(13)**

References

1. *APEX2, Version 2 User Manual, M86-E01078,*; Bruker Analytical X-ray Systems: Madison, WI, June 2006.

2. Sheldrick, G. M., SADABS (version 2008/1): Program for Absorption Correction for Data from Area Detector Frames, University of Göttingen, 2008.

3. Sheldrick, G. Crystal structure refinement with SHELXL. *Acta Crystallogr., Sect. A: Found. Crystallogr.* **2015**, *71*, 3-8.

4. Dolomanov, O. V.; Bourhis, L. J.; Gildea, R. J.; Howard, J. A. K.; Puschmann, H. *J. Appl. Crystallogr.* **2009**, *42*, 339-341.
